# Single-cell RNA-seq mapping of chicken peripheral blood leukocytes

**DOI:** 10.1186/s12864-024-10044-4

**Published:** 2024-01-29

**Authors:** Matilda Maxwell, Robert Söderlund, Sonja Härtle, Eva Wattrang

**Affiliations:** 1 Department of Microbiology, Swedish Veterinary Agency, Uppsala, Sweden; 2 Department for Veterinary Sciences, Ludwig-Maximilians-Universität Munich, Munich, Germany; 3 Present Address: Department of Clinical Sciences, Faculty of Medicine, Lund University, Lund, Sweden

**Keywords:** Chicken, Single-cell RNA-seq, Peripheral blood leukocytes

## Abstract

**Background:**

Single-cell transcriptomics provides means to study cell populations at the level of individual cells. In leukocyte biology this approach could potentially aid the identification of subpopulations and functions without the need to develop species-specific reagents. The present study aimed to evaluate single-cell RNA-seq as a tool for identification of chicken peripheral blood leukocytes. For this purpose, purified and thrombocyte depleted leukocytes from 4 clinically healthy hens were subjected to single-cell 3′ RNA-seq. Bioinformatic analysis of data comprised unsupervised clustering of the cells, and annotation of clusters based on expression profiles. Immunofluorescence phenotyping of the cell preparations used was also performed.

**Results:**

Computational analysis identified 31 initial cell clusters and based on expression of defined marker genes 28 cluster were identified as comprising mainly B-cells, T-cells, monocytes, thrombocytes and red blood cells. Of the remaining clusters, two were putatively identified as basophils and eosinophils, and one as proliferating cells of mixed origin. In depth analysis on gene expression profiles within and between the initial cell clusters allowed further identification of cell identity and possible functions for some of them. For example, analysis of the group of monocyte clusters revealed subclusters comprising heterophils, as well as putative monocyte subtypes. Also, novel aspects of TCRγ/δ + T-cell subpopulations could be inferred such as evidence of at least two subtypes based on e.g., different expression of transcription factors *MAF*, *SOX13* and *GATA3*. Moreover, a novel subpopulation of chicken peripheral B-cells with high *SOX5* expression was identified. An overall good correlation between mRNA and cell surface phenotypic cell identification was shown.

**Conclusions:**

Taken together, we were able to identify and infer functional aspects of both previously well known as well as novel chicken leukocyte populations although some cell types. e.g., T-cell subtypes, proved more challenging to decipher. Although this methodology to some extent is limited by incomplete annotation of the chicken genome, it definitively has benefits in chicken immunology by expanding the options to distinguish identity and functions of immune cells also without access to species specific reagents.

**Supplementary Information:**

The online version contains supplementary material available at 10.1186/s12864-024-10044-4.

## Background

The domestic fowl is the world’s most ubiquitous livestock and poultry products, meat and eggs, are an important source of protein for the growing human population. Moreover, the number of chickens reared worldwide is predicted to continue to increase [1, 2]. One vital part in safeguarding chicken health and welfare in all types of husbandry systems is to achieve effective and sustainable control of infectious diseases. To succeed in developing such measures comprehensive knowledge on the chicken immune system is paramount. Studies of the chicken immune system have historically contributed to several important findings even for mainstream immunology, e.g., the delineation of B- and T-cell lymphoid systems, but currently chicken immunology is lagging behind the mammalian counterparts particularly mouse and human immunology [3]. The general organisation of the chicken immune system is very similar to those of mammals, but several important differences exist, and it is thus vital not to indiscriminately translate new knowledge from mammals without verifying with chicken specific data. A large part of modern immunology involves in-depth phenotypic and functional identification of different cells of the immune system, e.g., T-cell subpopulations. Chicken immune cells can currently be phenotypically identified at protein level by immunofluorescence labelling and flow cytometric analysis using chicken specific reagents to several canonical immune markers, e.g., co-receptors CD4 and CD8, and some markers unique for chickens, e.g., the transmembrane protein Bu-1 (chB6) expressed on B-cells and some none-immune cells [4] and the mannose receptor MMR1L4 (MRC1L-B) expressed on monocytes [5, 6]. Indeed, in addition to identification of standard leukocyte populations such as B-cells, T-helper (Th) cells and cytotoxic T-cells (CTL) using these available reagents, various “special features” of chicken immune cells have also been discovered and novel rare/unusual immune cells have been indicated. For example, it was recognised early that chickens have a stable population of CD4 + CD8 αα+ T-cells in blood, spleen and among intestinal intraepithelial lymphocytes [7, 8]. Recently, presence of chicken TCRα/β + CD4 + CD8αβ+ [9] and TCRα/ β+ CD4-CD8αα + T-cells [10] was also suggested. Moreover, chickens belong to the group of species that have high numbers of TCRγ/δ + T-cells in the circulation and secondary lymphoid organs [11] and three main subpopulations of these have been identified so far [12]. However, work to further increase the resolution of chicken immune cell phenotyping is often hampered by dearth of chicken-specific reagents to detect crucial immunological markers such as cytokines and transcription factors for cell identification at protein level. In addition, it may also be difficult to assess which markers to focus reagent development efforts on without prior species-specific knowledge on their expression. Massively parallel single-cell RNA sequencing (RNA-seq) enables analysis of single cells with whole-transcriptome resolution without development of species-specific reagents provided that an appropriately annotated genome for the species in question exists. The methodology may then offer a higher dimensionality for analysing individual immune cells compared to e.g., flow cytometry with selected markers, and it may be possible to identify not only previously known but also novel cell types, to infer cell functions and to identify suitable cell markers [13].

The present study aimed to evaluate the use of single-cell RNA-seq for in-depth phenotypic and putative functional identification of chicken peripheral blood leukocytes. Single-cell RNA-seq has fruitfully been used to study mixed immune cell populations from several “non-mainstream” species such as pigs [14], horses [15], and teleost fish [16]. For chicken immune cells, purified “lymphocytes” [17], spleen cells [18] and bursal cells [19] have been used for single-cell RNA-seq but the focus of these studies have been on different viral infections and limited immune cell identification was reported. Peripheral blood is an easily accessible source of leukocytes but mRNA analysis of these in the chicken is complicated by presence of both nucleated red blood cells and thrombocytes. We therefore sought to decrease these cell types that were not the focus of the present investigation among cells submitted to sequencing. Chicken red blood cells can be reduced by Ficoll gradient centrifugation that in mammals is considered to enrich so called peripheral blood mononuclear cells (PBMC), mainly monocytes and lymphocytes. For chickens however, cells enriched by this procedure comprise a high proportion of thrombocytes that are of similar size as chicken lymphocytes. Chicken thrombocytes exclusively express the CD41/61 intergrin complex [20] and we used this as target for immunomagnetic separation to reduce the proportion of thrombocytes in the leukocyte preparations submitted for sequencing.

## Results

### Outcome of peripheral blood leukocyte isolation

For this study leukocytes were isolated from blood from four 24-week-old laying hens by Ficoll gradient centrifugation to reduce the content of red blood cells. Subsequently, the proportion of thrombocytes was reduced by immunomagnetic separation based on the expression of the intergrin complex CD41/61. Immunofluorescence phenotyping of the cell preparations showed that the proportion of thrombocytes was reduced from 46 to 60% CD41/61 + cells before depletion to 5–7% after (Additional file 1). Moreover, flow cytometric phenotyping (using antibody panels and gating strategies described in Additional files 5 and 6) showed that in the final depleted cell preparations submitted to sequencing, cells that were identified were on average 52% lymphocytes, 16% monocytes, 11% red blood cells and 6% heterophils.

### Defining the general single-cell resolution landscape of peripheral blood leukocytes by expression of marker genes

**Fig. 1:**
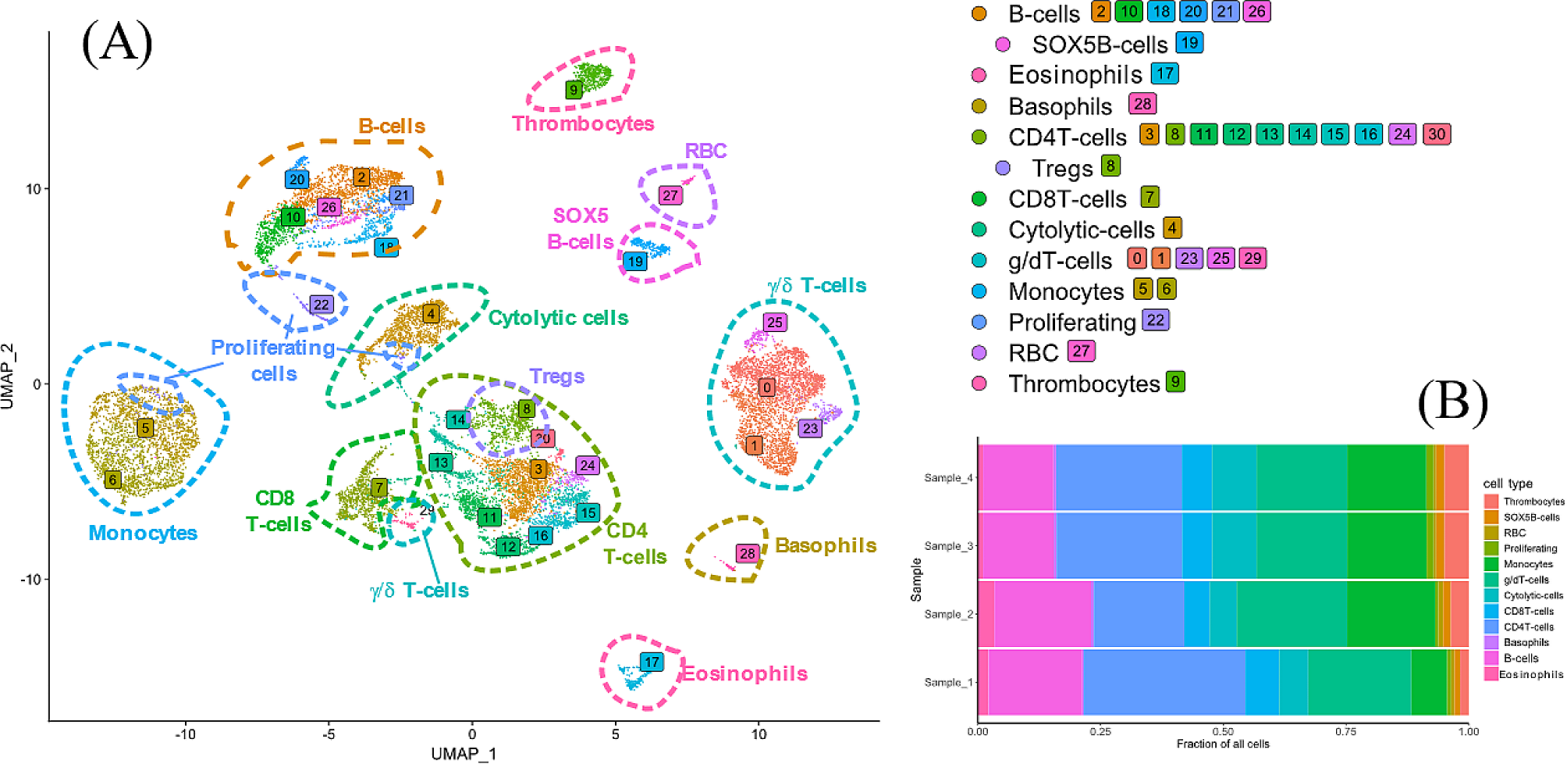
(**A**) UMAP visualization of the 16 936 studied cells in a 2D space after clustering with putative cell type annotations for the main clusters. Cells (points) are coloured based on their cluster identity. Putative cell types have been annotated using manual annotation from marker expression and gene ontology of expressed genes (Table 1). (**B**) Fractions of cell types for cell preparations from the individual hens

**Fig. 2:**
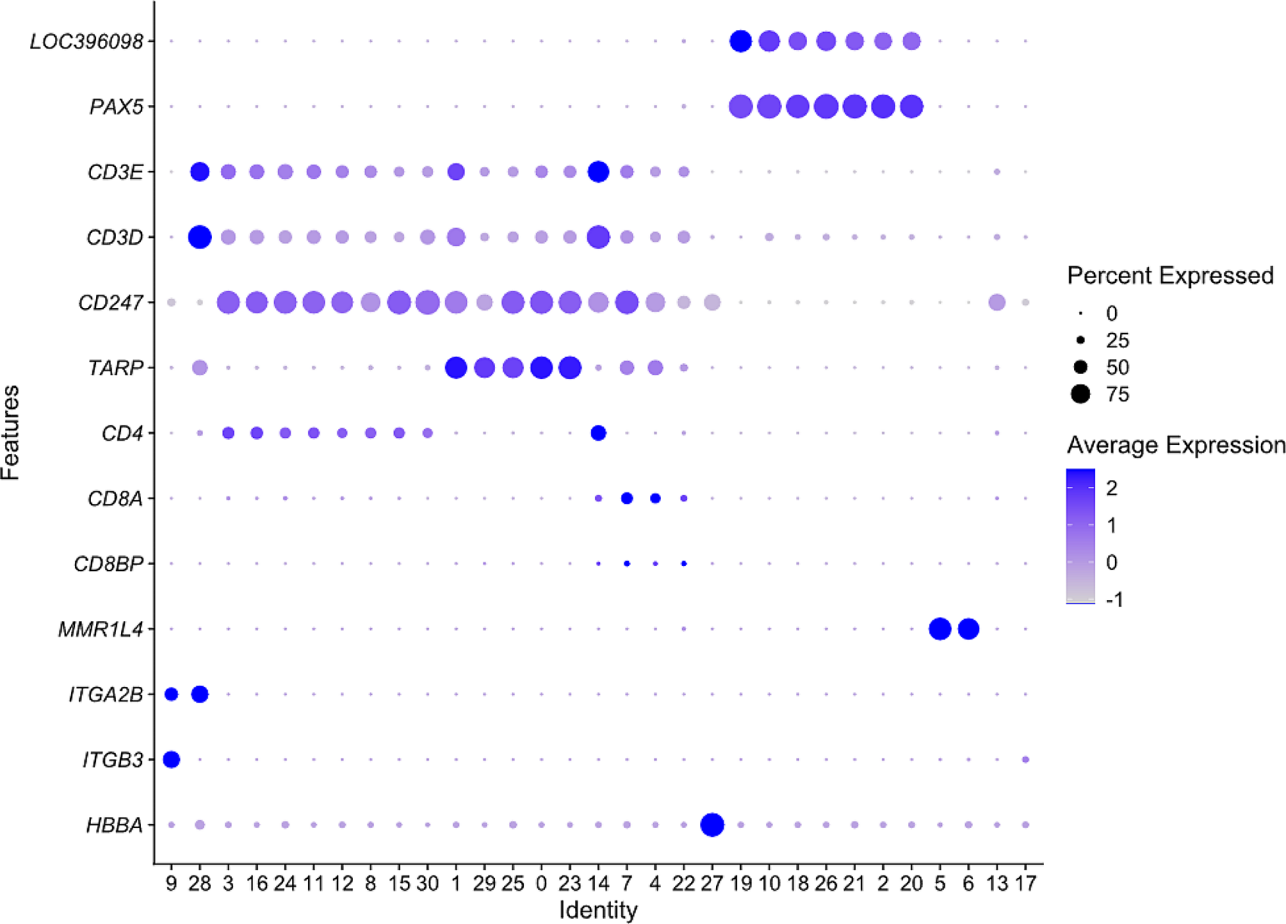
Dot plot of expression of marker genes (Features) in the indicated clusters (Identity) defined in Fig. 1. The radius of the dot corresponds to percentage of cells in each cluster expressing the gene, and colour intensity corresponds to scaled expression values. Expression values are scaled within the plot. *LOC396098* – Bu-1

Initial quality assessment from sequencing of the four samples showed that on average 4773 cells per sample (range 3020–5557 cells/sample) and 47,970 reads per cell (34,045–66,925 reads/cell) were analysed, the median number of detected genes per cell ranged from 1025 to 1202 and the sequencing saturation ranged from 84 to 91%. Unsupervised graph-based clustering of the total 16,936 cells that passed the final quality control, i.e., integrated from the four individual chickens, resolved 31 clusters (Fig. 1A). A selection of marker genes (Table 1) was used for cluster identification. These genes were either universally recognised leukocyte markers, e.g., CD3, chicken specific leukocyte markers, e.g., Bu-1, or identified as useful in the literature. Based on marker gene expression patterns and graph-based clustering on principal components, clusters were assigned to 11 groups with 2 subgroups; monocytes, B-cells (with subgroup *SOX5* + B-cells), TCRγ/δ T-cells, CD4 T-cells (with subgroup putative Treg), CD8 T-cells, “cytolytic cells”, putative basophils, putative eosinophils, proliferating cells, thrombocytes and red blood cells. All these cell groups were represented in similar proportions in the four individual chickens (Fig. 1B). The expression pattern of a selection of marker genes across the different clusters is shown in Fig. 2. Data for most clusters within the different groups were analysed in detail as described below. Cells in cluster 27 were identified as red blood cells based on the high expression of *HBBA* (Fig. 2) and cells in cluster 9 were identified as thrombocytes based on the expression of *ITGA2B* and *ITGB3* that in combination make the CD41/61 complex exclusive for chicken thrombocytes. These two clusters were not analysed further.

**Table 1:** Marker genes used for primary annotation of cell types among chicken leukocytes

Gene abbreviation	Name	Cell type	Comment
*LOC396098*	chB6/Bu-1	B-cell	Unique for chicken B-cells [4]
*PAX5*	Paired box 5	B-cell	
*CD247*	CD3 ζ subunit of T-cell receptor complex	T-cell	Also expressed in NK-cells [59]
*CD3E*	CD3ε subunit of T-cell receptor complex	T-cell	
*CD3D*	CD3δ subunit of T-cell receptor complex	T-cell	
*TARP*	TCR gamma alternate reading frame protein	TCRg/d + T-cell	
*CD4*	Cluster of differentiation 4	T-helper cell (Th)	
*CD8A*	Cluster of differentiation 8α	T-cell	In chickens expressed on several T-cell types and some NK-cells [71]
*CD8BP*	Cluster of differentiation 8β pseudogene	T-cell	In chickens expressed on CTL and some TCRg/d + T-cells [12]
*MMR1L4*	macrophage mannose receptor 1-like 4	monocyte	Exclusively expressed on chicken monocytes [5, 6]
*ITGA2B*	integrin subunit alpha 2β / CD41	thrombocyte	The CD41/61 complex exclusively expressed on chicken thrombocytes [20]
*ITGB3*	integrin subunit beta 3 /CD61	thrombocyte	The CD41/61 complex exclusively expressed on chicken thrombocytes [20]
*HBBA*	hemoglobin subunit epsilon 1	red blood cell	
*MMP9*	Matrix metallopeptidase 9	heterophil	Expressed in chicken heterophils but not in chicken monocytes [40]
*GNLY*	Granulysin	Cytolytic cell	
*FASLG*	Fas ligand	Cytolytic cell	
*GZMA*	Granzyme A	Cytolytic cell	
*GZMM*	Granzyme M	Cytolytic cell	
*IL2RA*	Interleukin-2 receptor subunit α	T-regulatory cell (Treg)	Among resting CD4 + T-cells primarily expressed in Treg [54]
*CTLA4*	Cytotoxic T-lymphocyte associated protein 4 (CD152)	Treg	Among resting CD4 + T-cells primarily expressed in Treg [54]
*MKI67*	Marker of proliferation Ki-67	Proliferating cell	Expression associated with cell proliferation [72]
*PPIA*	Peptidylprolyl isomerase A	Proliferating cell	Expression associated with cell proliferation [73]
*YBX1*	Y-box binding factor 1	Proliferating cell	Expression associated with cell proliferation [74, 75]
*DACH1*	Dachshund family transcription factor 1	Eosinophil	Eosinophil associated gene [76]
*MCTP2*	Multiple C2 and transmembrane domain containing 2	Eosinophil	Eosinophil associated gene [76]
*PIP5K1B*	Phosphatidylinositol-4-phosphate 5-kinase type-1 beta	Eosinophil	Eosinophil associated gene [76]
*FRY*	FRY microtubule binding protein	Eosinophil	Eosinophil associated gene [76]
*LOC770612*	interferon-induced transmembrane protein 1-like (IFITM1)	Eosinophil	Eosinophil associated gene [76]
*IL5RA*	Interleukin-5 receptor subunit α	Eosinophil	Eosinophil associated gene [76]
*CTSG*	Cathepsin G	Granulocyte, basophil, mast cells	Serine protease expressed in granulocytes including basophils and in mast cells [77]
*HDC*	Histidine decarboxylase	Basophil	Basophil/mast cell associated gene [78]
*GATA2*	GATA-binding factor 2	Basophil	Basophil/mast cell associated gene [79]
*FCER1G*	Fcε receptor Ig	Basophil	Expressed mainly on basophils/mast cells but also on e.g., eosinophils [80]
*NDST2*	N-deacetylase and N-sulfotransferase 2	Basophil	Basophil/mast cell associated gene [78]
*CD63*	Cluster of differentiation 63	Basophil	Expressed on basophils [81]

### Monocyte clusters and sub-cluster analysis

**Fig. 3:**
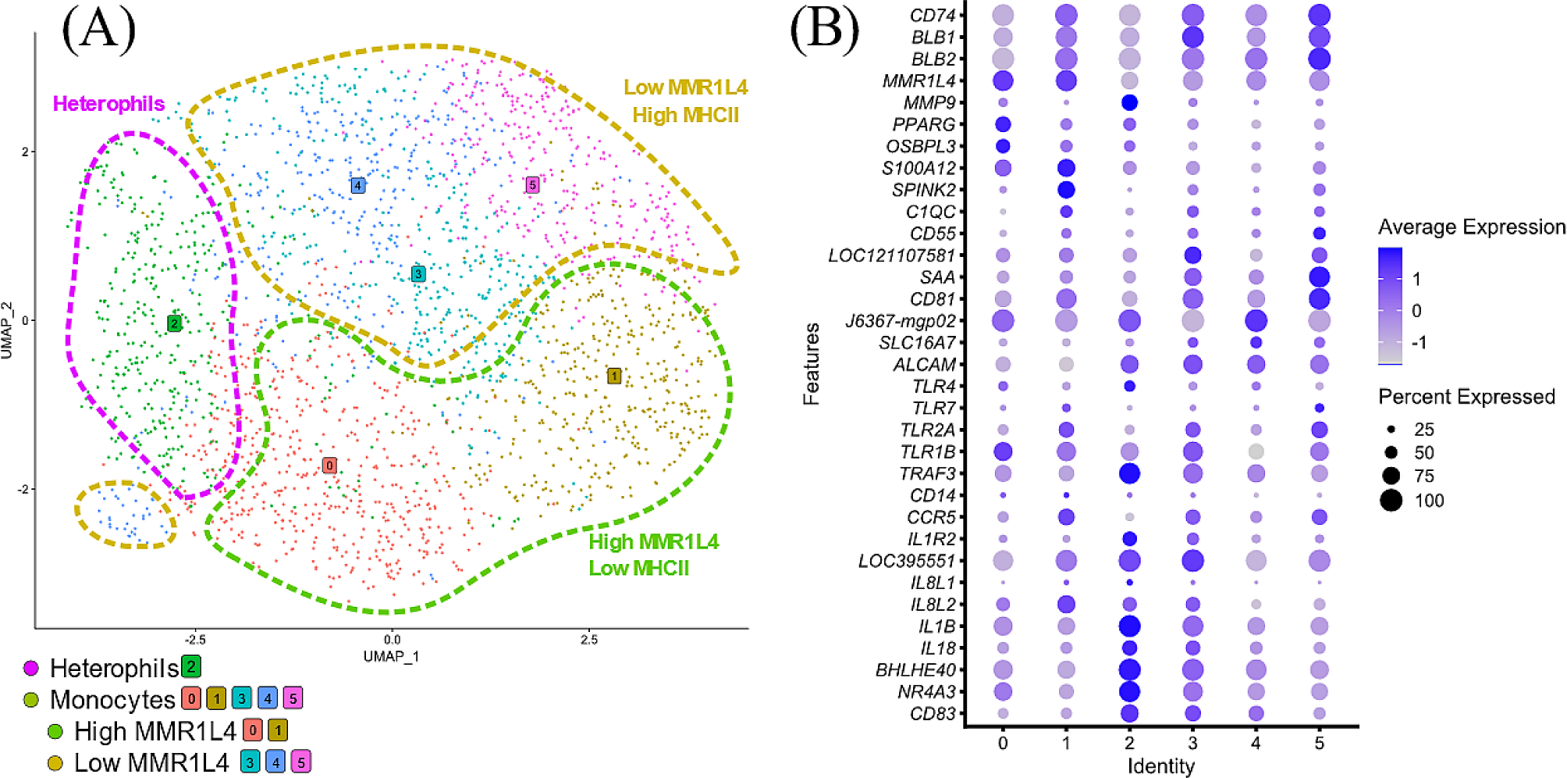
(**A**) UMAP after re-clustering of cells in initial monocyte clusters 5 and 6 (*MMR1L4* + cells) at resolution 0.8. The analysis identified six subclusters 0–6 identified by different colour cells. Subclusters identified as heterophils, low MMR1L4 high MHCII expressing and high MMR1L4 low MHCII expressing, respectively are indicated. (**B**) Dot plot of expression of a selection of monocyte and heterophil associated genes in the indicated subclusters. The radius of the dot corresponds to percentage of cells in each cluster expressing the gene, and colour intensity corresponds to scaled expression values. Expression values are scaled within the plot. *LOC121107581* – S100-A4-like, *J6367-MGP02*– CYTB, *LOC395551*– CCL4

The distinct group formed by clusters 5 and 6 (Fig. 1A) was defined by expression of *MMR1L4* (Table 1; Fig. 2) as mainly comprising monocytes. Further analysis of gene expression within clusters 5 and 6 (Additional file 2) showed that relative expression of *MMR1L4* and MHCII genes *BLB1, BLB2*, and *CD74* differed between the clusters revealing a pattern of high *MMR1L4*/low MHCII expression in cluster 5 and low *MMR1L4*/high MHCII expression in cluster 6. Moreover, expression of *MMP9* was noted in some cells in cluster 6 which suggested this cluster in addition to monocytes also could comprise heterophils. Therefore, a re-cluster analysis was performed on clusters 5 and 6 in which six sub-clusters (0–5) were indicated (Fig. 3A). Based on the differential expression of *MMR1L4*, exclusively expressed in chicken monocytes, MHCII genes and *MMP9* that is expressed in chicken heterophils but not in chicken monocytes, we found that sub-clusters 0, 1, 3, 4, and 5 comprised mainly monocytes and that sub-cluster 2 likely contained mainly heterophils (Fig. 3B). Moreover, the re-clustering also revealed a polarisation of cells with high *MMR1L4* expression and low/lower MHCII expression (cluster 0,1) and low *MMR1L4* expression and high/higher MHCII expression (cluster 3,4,5), as seen in the original clustering. Differential expression of a selection of immune/immune function genes (Fig. 3B) indicate differences in functions and monocyte phenotype as discussed below. The top enriched GO- and KEGG-terms (List at Gene Expression Omnibus (GEO); www.ncbi.nlm.nih.gov/geo, accession number GSE224329) for original cluster 5 comprised several terms concerning MHCII and antigen presentation on MHCII and for cluster 6 different terms for chemotaxis and cell migration were prominent. The top enriched GO and KEGG terms for sub-clusters comprised chemotaxis and myeloid leukocyte migration for subclusters 0, 1 and 3, Toll-like and NOD-like receptor signalling for subcluster 2, myeloid leukocyte differentiation for subclusters 4 and 5, and MHC II for subclusters 1 and 5.

### B-cell clusters

**Fig. 4:**
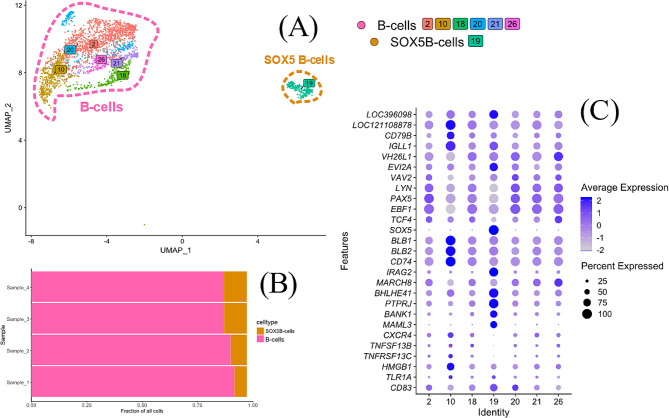
(**A**) UMAP for B-cells (*LOC396098*+, i.e., Bu-1+) with putative cell types established based on the differential expression of Bu-1 within the data. (**B**) Fractions of B-cell types for cell preparations from the individual hens. (**C**) Dot plot of expression of a selection of B-cell associated genes in the B-cell clusters. The radius of the dot corresponds to percentage of cells in each cluster expressing the gene, and colour intensity corresponds to scaled expression values. Expression values are scaled within the plot. *LOC121108878* - CD79A, *LOC395551*– CCL4

B-cells (clusters 2, 10, 18, 19, 20, 21 and 26; Fig. 4) were primarily identified by the expression of the chicken specific B-cell marker Bu-1, *LOC396098*, as well as B-cell master regulator transcription factor *PAX5* (Table 1; Fig. 2). Clusters 2, 10, 18, 20, 21 and 26 formed a distinct group of clusters while cluster 19 was positioned on its own (Fig. 4A). Differential expression of a selection of B-cell associated genes and other immune genes was analysed for the B-cell clusters (Fig. 4C). This showed that all these clusters had a high expression of other typical B-cell associated genes such as the B-cell receptor genes CD79A (*LOC121108878*) and *CD79B*, immunoglobulin light chain, *IGLL1*, and IgA, *VH26L1*, B-cell transcription factors *EBF1* and *TCF4*, and MHCII genes *BLB1*, *BLB2* and *CD74*. The expression pattern of the selected genes was similar between these clusters except for cluster 19 and cluster 10. Of the latter two, cells in cluster 19 showed a high expression of *SOX5*, which was unique among the B-cell clusters. In addition, cells in cluster 19 showed the highest expression of Bu-1, *EVI2A*, *IRAG2*, *BHLHE41*, *PTPRJ*, *MAML3* and *BANK1* among the B-cell clusters. In contrast, cells in cluster 10 showed the highest expression of B-cell receptor genes *CD79A* and *CD79B*, *IGLL1*, MHCII genes, *CXCR4*, BAFF (*TNFSF13B*), BAFF-receptor (*TNFRSF13C*) and *HMGB1* among the B-cell clusters. Cells in cluster 10 also showed a high expression of different ribosomal proteins and the top enriched GO- and KEGG-terms (Lists at GEO; GSE224329) for this cluster involve ribosomes but also GO-terms concerning MHC and antigen processing and presentation on MHCII were identified for this cluster. For clusters 2, 18, 19, 20, 21 and 26 different B-cell and immunoglobulin associated terms as well as C-type lectin receptor signalling pathway were among the highest enriched GO- and KEGG-terms (List at GEO; GSE224329). For cluster 19, Negative regulation of response to oxidative stress (GO:1,902,883) was also enriched.

### T-cell clusters

**Fig. 5:**
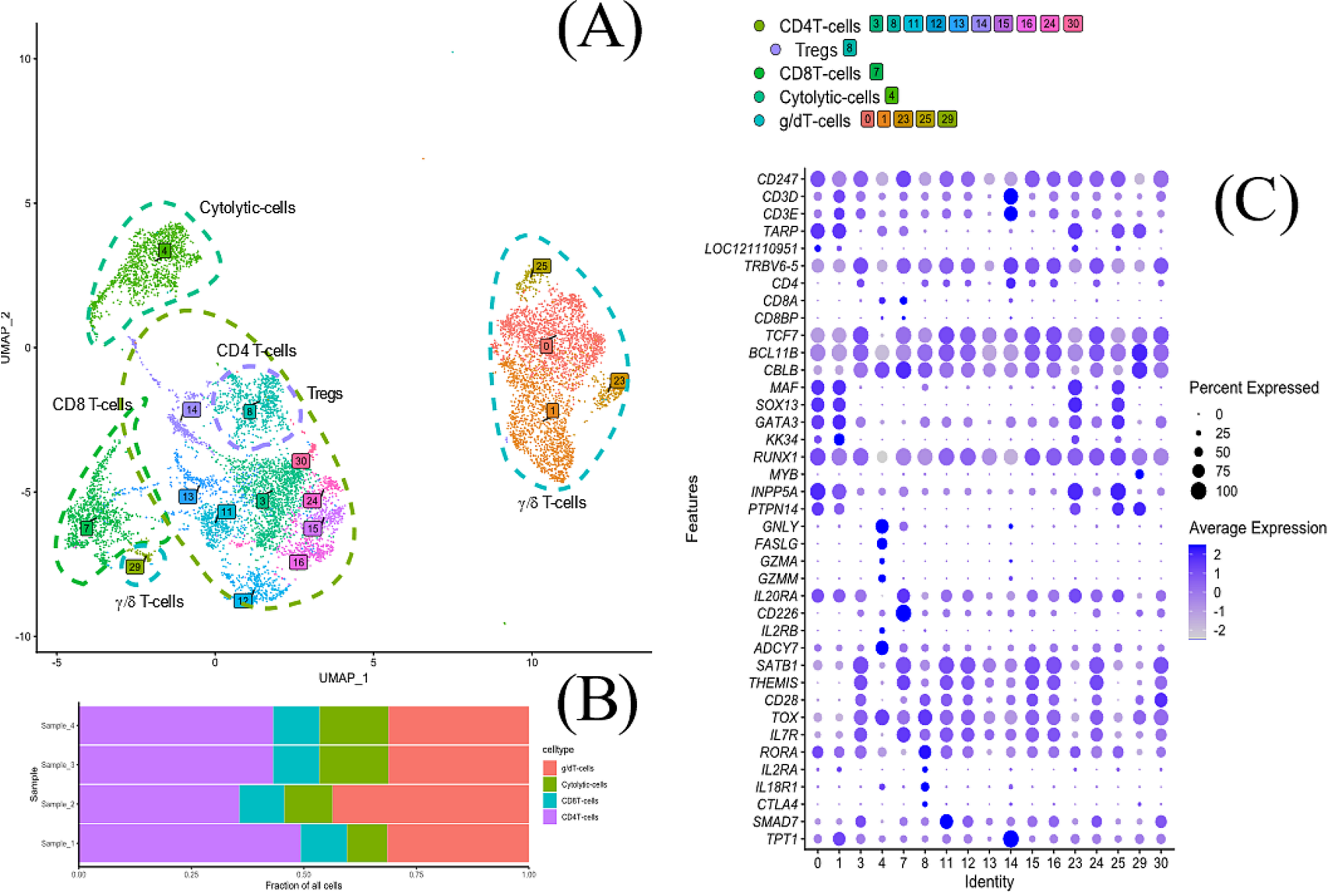
(**A**) UMAP for a subset of data corresponding to T-cells with putative T-cell types annotated based on the differential expression within the subcluster. (**B**) Fractions of T-cell types for cell preparations from the individual hens. (**C**) Dot plot of expression of a selection of T-cell associated genes in the T-cell clusters. The radius of the dot corresponds to percentage of cells in each cluster expressing the gene, and colour intensity corresponds to scaled expression values. Expression values are scaled within the plot. *LOC121110951* - TCR δ-chain

Clusters 0, 1, 3, 4, 7, 8, 11–16, 23, 24, 25, 29, and 30 (Fig. 5) were annotated as T-cells based on their expression of T-cell marker genes *CD247*, *CD3E*, *CD3D*, *TARP*, *CD4*, *CD8A* and *CD8BP* (Table 1; Fig. 2). These clusters (Fig. 5A) were analysed for differential expression of a selection of T-cell associated genes and other immune genes (Fig. 5C). Cells in cluster 4 were then, based on expression of genes associated with cytolytic functions, *GNLY*, *FASLG*, *GZMA* and *GZMM*, attributed cytolytic capacity and this cluster is presented separately in the section below. The remaining T-cell clusters were assigned as containing mainly: TCRγ/δ + T-cells: 1, 0, 23, 25 and 29; CD4 + cells: 3, 8, 11, 12, 13, 14, 15, 16, 24 and 30 and CD8αβ+ cells: 7.

#### TCRγ/δ + T-cells

Cluster 1, 0, 23 and 25 formed a distinct group of clusters while cluster 29 grouped together with cluster 7, i.e., CD8αβ+ cells (Fig. 5A). Cells in clusters 1, 0, 23 and 25 were identified by high expression of *TARP* and also showed expression of the TCR δ-chain (*LOC121110951*; Fig. 5B). Among the selection of T-cell associated and other immune genes, cells in these clusters also shared a high expression of *MAF*, *SOX13*, *GATA3*, *KK34* and *INPP5A* that distinguished them from the other T-cell clusters (Fig. 5C). Cells in all TCRγ/δ+ T-cell clusters also showed a low expression of *IL7R* compared to cells in CD4 + and CD8αβ+ T-cell clusters. Apart from a high expression of *TARP* cells in cluster 29 showed a different expression pattern compared to the other TCRγ/δ + T-cell clusters but shared the expression of *PTPN14* with these, which was distinct from other T-cell clusters. Cells in cluster 29 also showed a distinct expression of *MYB* and a high expression of *BCL11B* compared to other T-cell clusters and high expression of *CBLB* similar to that observed for cluster 7. For cluster 0 the top enriched GO-terms involved nucleic acid transcription, and for clusters 23 and 25 regulation of nucleic acid transcription (List at GEO; GSE224329). Cells in cluster 1 showed a high expression of different ribosomal proteins (List at GEO; GSE224329) and the top enriched GO- and KEGG-terms for this cluster involve ribosomes. For cluster 29 top enriched GO-terms included those involved in T-cell activation, T cell differentiation and T-cell receptor recombination.

#### CD4 + T-cells

Clusters 3, 8, 11–16, 24 and 30 formed a group of clusters in close proximity of the group of clusters 7 (CD8αβ + cells) and 29 (TCRγ/δ + T-cells, Fig. 5A). All these clusters contained some cells that expressed *CD4*, albeit very few in cluster 13 (Fig. 5B). With respect to expression of the selected T-cell associated and other immune genes the expression pattern of most of these clusters was similar (Fig. 5C). However, in cluster 8 some cells showed a high expression of *IL2RA* (CD25) and *CTLA4* and this cluster was therefore putatively identified as comprising regulatory T-cells (Treg). Distinctive for cluster 8 was also some cells with high expression of *IL18R1* and the relatively highest expression of *RORA* compared to other T-cell clusters. For clusters 3, 8, 11, 12, 13, 15, 16, 24 and 30, GO- and KEGG-terms concerning different aspects of T-cells such as T cell activation, CD4-positive, alpha-beta T cell activation and T cell receptor signalling pathway were among the top enriched (List at GEO; GSE224329). Cells in cluster 14 showed the relatively highest expression of *CD3D*, *CD3E* and *CD4* and had a distinctive high expression of *TPT1* among the T-cell clusters, and also comprised some cells expressing *CD8A*, *CD8BP* and *GNLY*. Cells in this cluster showed a high expression of different ribosomal proteins (List at GEO; GSE224329) and the top enriched GO- and KEGG-terms involve ribosomes.

#### CD8αβ + T-cells

Cluster 7 grouped with cluster 29 (Fig. 5A) and contained cells with high expression of CD8A and CD8BP (Fig. 5C) and was therefore identified as comprising CD4-CD8αβ+ T-cells Among the selected T-cell associated and other immune genes, cells in cluster 7 showed a distinct high expression of *CD226*. The top enriched GO- and KEGG-terms for this cluster involved immune response activation and signalling, T-cell receptor signalling and T-cell receptor recombination (List at GEO; GSE224329).

### Cytolytic cell cluster and sub-cluster analysis

**Fig. 6:**
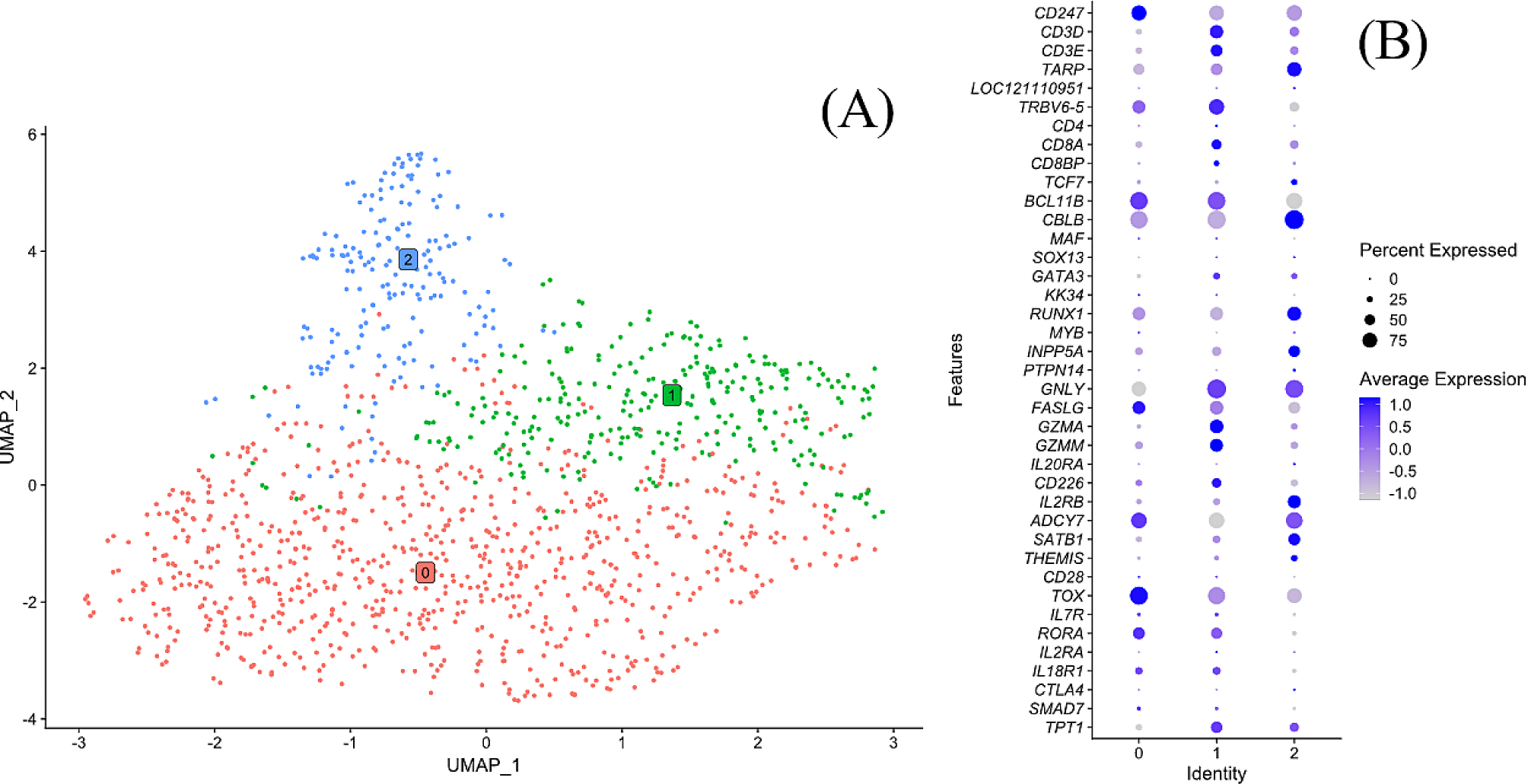
(**A**) UMAP after re-clustering cells of cytolytic cells in initial cluster 4 with putative cell types annotated based on the differential expression within the subcluster. The analysis identified three subclusters 0–2 identified by different colour cells. (**B**) Dot plot of expression of a selection of T-cell associated genes in the cytolytic subclusters. The radius of the dot corresponds to percentage of cells in each cluster expressing the gene, and colour intensity corresponds to scaled expression values. Expression values are scaled within the plot. *LOC121110951* - TCR δ-chain

Cluster 4 was positioned on its own in proximity to the CD4+ and CD8αβ+ T-cells (Fig. 5A). Cells in cluster 4 were judged as comprising T-cells based on the expression of T-cell marker genes (Table 1; Fig. 2) and also showed a high expression of genes associated with cytolytic functions, *GNLY*, *FASLG*, *GZMA* and *GZMM* (Fig. 5C). Some cells in this cluster showed a strong expression of *CD8A* and *CD8B* but the expression of *TARP*, *TRVB6-5* (TCRβ), *CD247*, *CD3D* and *CD3E* was relatively low. Therefore, cluster 4 was annotated as cells with cytolytic capacity of probably mixed lineage and a re-cluster analysis was performed to investigate cell types within the cluster (Fig. 6). This analysis indicated three subclusters (0–2; Fig. 6A). Cells in sub-cluster 0 constituted approximately 50% of cells in the original cluster 4 and showed low expression of *CD3D*, *CD3E*, *TARP* and *TRBV6-5* indicating that this sub-cluster comprised low numbers of T-cells while high expression of *CD247* (CD3ζ) suggests that a majority of cells might be NK cells. Of the studied genes indicative of cytolytic activity, cells in sub-cluster 0 showed a high expression of *FASLG*. Cells in this sub-cluster also showed a distinct high expression of *TOX*, as well as high expression of *BCL11B* and *ADCY7*. Cells in sub-cluster 1 showed a high expression of *CD3D*, *CD3E* and *TRBV5-6* as well as *CD8A* and *CD8BP*, indicating that this sub-cluster contained CTL. In this sub-cluster all the studied genes indicative of cytolytic activity showed significant expression and *GZMA* and *GZMM* had the highest expression and *FASLG* the lowest. Cells in sub-cluster 1 also showed high expression of *BCL11B* and some cells showed a high expression of *CD226*. Cells in sub-cluster 2 expressed high levels of *TARP*, indicating that it contained TCRγ/δ + T-cells. Of the genes indicative of cytolytic activity, *GNLY* showed the highest expression in this sub-cluster. Cells in this sub-cluster also showed a high expression of *CBLB*, *RUNX1*, *INPP5A*, *IL2RB*, *ADCY7* and *SATB1*. For cluster 4 as well as sub-clusters 0–2, top enriched GO- and KEGG-terms involved activation of leukocytes and different lymphocyte malignancies (Lists at GEO; GSE224329).

### Proliferating cell cluster

Cluster 22 likely consisted of proliferating cells as indicated by their high expression of proliferation-associated genes e.g., *MKI67*, *PPIA* and *YBX1* (Table 1) and many ribosomal proteins (List at GEO; GSE224329). Some cells assigned to cluster 22 formed clusters of cells within groups belonging to three other primary cell types: cytolytic cells in cluster 4, monocytes in cluster 5 and 6, and B-cells in mainly cluster 10, while some cluster 22 cells form a distinct cluster not embedded in another cell population (Additional file 3 A). When re-clustered, these cells again form four clusters (0–3; Additional file 3B). Of these sub-clusters, cells in sub-cluster 0 remained fairly anonymous with the top expressed genes being involved in e.g., proliferation, mitosis and DNA transcription (List at GEO; GSE224329). However, due to this sub-cluster’s closeness to sub-clusters 2 and 3 and some expression of *CD4* and *CD28*, we suggest these cells were lymphocytes. For cells in sub-cluster 2 the top-10 expressed genes included *GNLY*, *GZMA*, *FASLG*, *CBLB*, *TARP* and *SH2D1A*, and several other cytolytic and CD3 genes as well as *CD8A* were also highly expressed, which indicated that this sub-cluster contained cytolytic cells including cytolytic TCRγ/δ + T-cells. For cells in sub-cluster 3 the top-10 expressed genes included *VH26L1* (IgA), *IGLL1*, *JCHAIN, MZB1*, and *PDIA4*, and several other typical B-cell genes were highly expressed, which indicated that this subcluster contained B-cells including, based on the expression of *JCHAIN*, plasma blasts. For cells in sub-cluster 1 the top-10 expressed genes included *IFI30*, *CD74*, *BLB2*, *LY86* and *CSF3R*, and several other genes associated with antigen presentation and myeloid cells including *MMR1L4* were highly expressed, which indicates that this sub-cluster comprised antigen presenting myeloid cells.

Enriched GO- and KEGG terms for cluster 22 included terms that are ribosome, cytoskeleton, and translation associated (List at GEO; GSE224329). Therefore, this cluster is likely a mixture of cells from different major cell groups that were actively proliferating.

### Putative eosinophil cluster

Cluster 17 was distinct from other groups (Fig. 1A) and cells in this cluster lacked strong expression of any of the general marker genes used for identification (Fig. 2). Based on high expression of *DACH1*, *MCTP2*, *PIP5K1B*, *FRY*, *LOC770612* (*IFITM1*), *IL5RA* and *FCER1G* (List at GEO; GSE224329) that have been associated with eosinophils (Table 1) this cluster was putatively assigned as eosinophils.

### Putative basophil cluster

Cluster 28 was distinct from other groups (Fig. 1A) and based on high expression of *CTSG*, *HDC*, *GATA2*, *FCER1G*, *NDST2* and *CD63* that are genes associated with basophils/mast cells (Table 1) as well as the KEGG-term Glycosaminoglycan biosynthesis - heparan sulfate / heparin (KEGG:00534; List at GEO; GSE224329) that was highly enriched for this cluster it was putatively assigned as basophils. However, genes normally associated with other cell types e.g., *CD3D* and *CD3E* (Fig. 2), were also highly expressed by cells in this cluster and it may thus contain a mixture of cell types.

### Correlation between cellular identification by mRNA expression and cell surface marker phenotyping

**Fig. 7:**
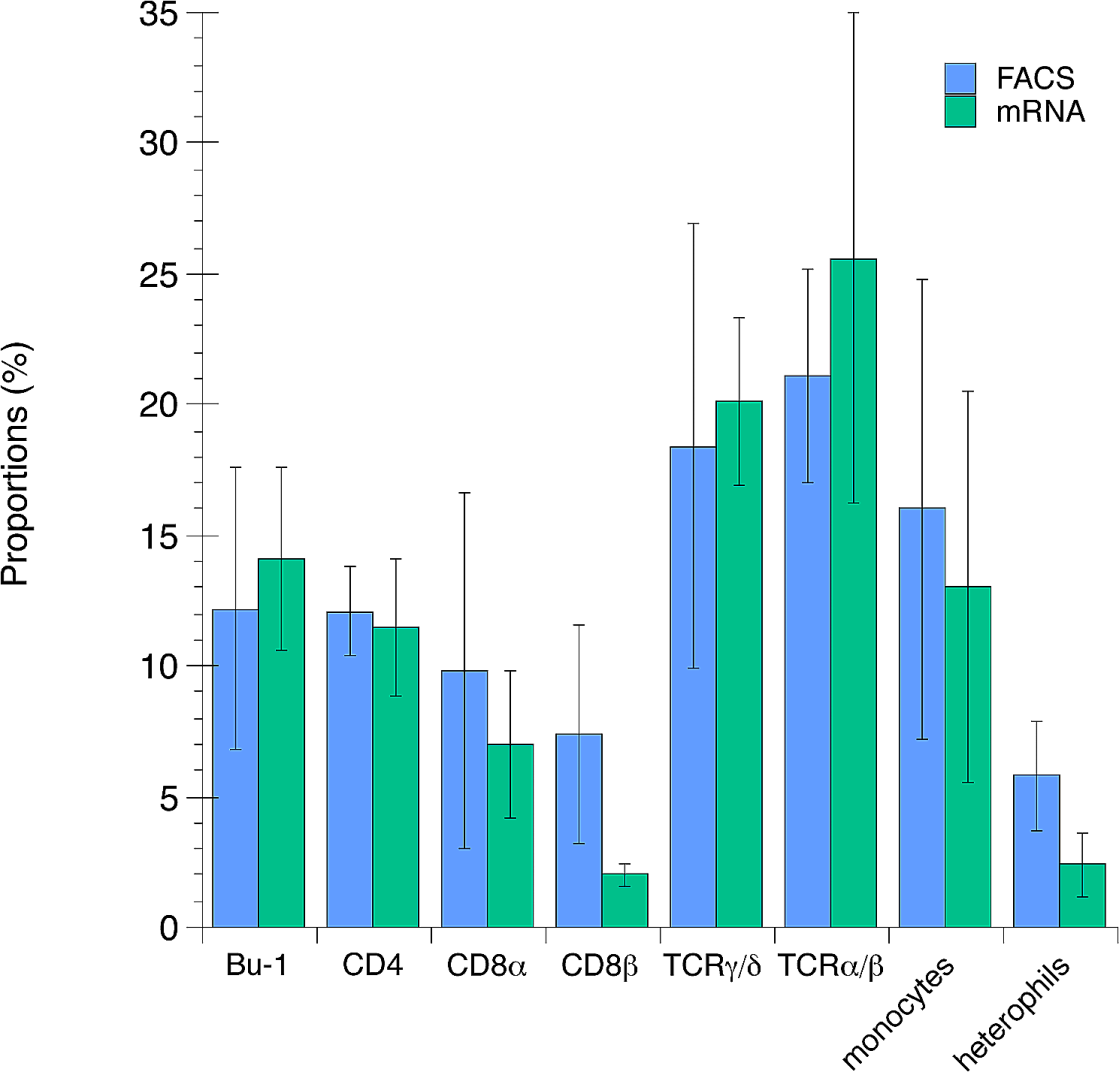
Identification of chicken leukocytes in the indicated populations by phenotypical identification by immunofluorescence labelling and flow cytometric analysis (FACS; blue bars) and by single-cell mRNA expression (mRNA; pink bars). For phenotypical identification, cells were identified as cells in the “lymphocyte gate” with cell surface expression of Bu-1, CD4, CD8α, CD8β, TCRγ/δ and TCRα/β (combination of TCRα/Vβ_1_ and TCRα/Vβ_2_), respectively. Monocytes were identified by FSC/SSC characteristics and cell surface expression of MMR1L4 (MRC1L-B) and heterophils were identified by FSC/SSC characteristics and cell surface expression of CD45. Data was expressed as proportions of populations out of live events for each individual hen. For gating strategies see Additional file 6. For mRNA identification, cells with log^2^ fold change ≥ 0.25 expression of *LOC396098* (Bu-1), *CD4*, *CD8A* (CD8α), *CD8BP* (CD8β) and *MMR1L4* were considered positive. For TCRγ/δ, cells in clusters 1, 0, 23, 25 and 29 were considered positive, for TCRα/β cells in clusters 3, 8, 11, 12, 13, 14, 15, 16, 24, 30 and 7 were considered positive and for heterophils cells in monocyte subcluster 2 were considered positive. Data was expressed as proportions of positive cells out of the total number of cells after filtering for each individual hen. Results shown are means ± 95% confidence intervals, *n* = 4

Results from the single-cell RNA-seq based identification of gene expression and cell identification were compared to phenotypical identification by immunofluorescence labelling and flow cytometric analysis overall (Fig. 7) and for the individual animals (Additional file 4). In general, the results from the two methods showed a good correlation. For CD8β + cells and heterophils mRNA expression values were on average lower than corresponding values from cell surface phenotyping.

## Discussion

This study was carried out as a guide to assess benefits and limitations of single-cell RNA-seq in phenotypic and functional identification of chicken leukocytes. Unsupervised graph-based clustering of single-cell RNA-seq data from peripheral blood leukocytes delineated the cell transcriptomes into 31 clusters. Employing the current annotation of the chicken genome it was possible to identify 28 of these with reasonable certainty by manual curation identifying expression of basic canonical marker genes and/or chicken specific immune genes. In addition, the remaining three clusters were putatively identified by recognition of gene expression patterns that could be compared to those of mammalian leukocytes. Moreover, the relative distribution of leukocyte populations defined by mRNA expression showed a good correlation to that identified by cell surface protein expression and flow cytometric characterisation. Hence, we were convinced that the identification of major leukocyte populations e.g., monocytes, heterophils, B-cells and different T-cells, was satisfactory. With respect to numbers and types of clusters identified and resolution of the identification, our results were also comparable to what has been reported for PBMC from horses (31 clusters [15]) and pigs (36 clusters [14]). In contrast, a study of single-cell RNA-seq of purified chicken “lymphocytes” reports a total of merely nine clusters and only three of these were identified, two T-cell clusters and one B-cell cluster [17]. We believe that differences in the bioinformatic analysis of data were the most likely reasons to the disagreeing outcome of that study compared to the present results even though they also report lower number of reads per cell compared to the current study. Differences in bioinformatic analyses between the two studies include stringency of filtering as well as choice of normalisation algorithm when normalising UMI counts. In the current study, the SCT transform normalisation method that has been specifically developed for single cell data in order to preserve biological heterogeneity [21] was used while Qu et al. [17] used the log-normalisation. Hafemeister and Satija [21] showed that the use of the SCT transform instead of the log-transform for normalisation of UMI count revealed additional biological substructure in several cell populations. We therefore believe that this difference in normalisation method could have impacted the interpretation of results between these two studies. Moreover, a study of single-cell RNA-seq of chicken spleen cells reports 12 clusters that were identified as three general T-cell clusters, two TCRγ/δ T-cell enriched clusters, two B-cell clusters, two macrophage clusters, two granulocyte clusters and one cluster of antigen presenting myeloid cells [18]. This latter study states limited resolution as a reason for not being able to identify different cell types, particularly T-cells, further. In comparison, the latter study reports that approximately 50% less genes were detected per cell [18] compared to the present results, which possibly explain their poor detection of genes with low expression levels e.g., CD4. Thus, the sequencing depth, numbers of analysed cells and bioinformatic approach of the current study generated considerably more information on leukocyte subtypes compared to these previous reports on chicken leukocytes and can probably be considered a “minimal requirement” for useful leukocyte subtype phenotyping by single-cell RNA-seq. Nonetheless, in some cases e.g., regarding expression of CD4 and CD8 genes, it seemed a further increased sequencing depth could potentially improve the resolution of identified cell subtypes. However, low gene expression of key transcripts for leukocyte phenotyping e.g., of T-cells, is a recognised problem in single-cell RNA-seq and other strategies such as changing the methodology for library preparation might also be needed to increase the ability of resolving these phenotypes by single-cell RNA-seq [22].

### B-cells

Among the leukocyte clusters in the current study, B-cell clusters were among the easiest to identify. Cells in the seven B-cell clusters showed strong expression of a whole panel of typical B-cell genes including transcription factors, B-cell receptor genes, immunoglobulin genes and Bu-1. Despite the ease of identification as B-cells, based on RNA-expression patterns for five of these clusters we were not able to distinguish between them and designate their identity or function beyond this. Nonetheless, cells in B-cell cluster 10 showed higher expression of B-cell receptor genes, MHC II genes, *IGLL1*, *CXCR4*, BAFF (*TNFSF13B*), BAFF-receptor (*TNFRSF13C*) and *HMGB1* compared to the other B-cell clusters, and they also showed a high expression of ribosomal proteins. We therefore concluded B-cells in this cluster could be designated as “activated”, i.e., active in protein synthesis and antigen presentation, which was also supported by the GO-terms enriched for this cluster.

B-cells in cluster 19 were positioned separately from the group formed by the other B-cell clusters indicating that these cells had an overall more distinctive RNA expression pattern than the other identified B-cells. The highest expressed gene in this cluster was *SOX5* which was not expressed in the other B-cell clusters. In human B-cells, high *SOX5* expression has been shown during late stages of differentiation [23] and the GO-term “negative regulation of response to oxidative stress” enriched for this cluster also supports terminal/late-stage differentiation of these cells [24]. In so-called CD11c + atypical B-cells (ABCs, [25]) high *SOX5* expression is a common feature. These ABC B-cells constitute a heterogeneous population with so far largely unknown functions that has been identified in humans, mice [25] and recently also among horse PBMC [15]. However, for chicken B-cells in the current cluster 19 we did not observe expression of other genes commonly identified for ABCs using single-cell RNA-seq such as *ITGAX* (CD11c), *TBX21* (T-bet), *FCRL4*, *FCRL5*, *ZEB2* or *FGR* [25]. Nevertheless, we observed increased expression of *BANK1* and *BHLHE41* which was likewise observed for horse T-bet + B-cells [15] and *BANK1* has been associated with human and murine ABCs [26, 27]. Additionally, cells in cluster 19 had a high expression of *BHLHE41*, *PTPRJ* and *MAML3* that in mammals have been associated with B-1 B-cells [28, 29] and marginal zone B-cells [30]. Neither ABC B-cells nor native type B-cells have yet been described in chickens but it is known that chickens have circulating natural antibodies [31] that in mammals are produced by native type B-cells such as B1 B-cells and marginal zone B-cells. Hence, based on the current knowledge on gene expression in chicken peripheral B-cells we cannot conclusively identify the B-cells in cluster 19 but suggest they are a distinct population of late stage differentiated B-cells of either “ABC” or native type that are a novel observation in chickens.

### Myeloid cells

To identify monocytes we used expression of *MMR1L4* (MRC1L-B), a member of the chicken mannose receptor family that is exclusively expressed in chicken monocytes/macrophages and corresponding to the mammalian mannose receptor C-type 1 (MRC1, CD206) [5, 6]. By *MMR1L4* expression, we initially identified clusters 5 and 6 that form a distinct separate group, as comprising monocytes. Further sub-cluster analysis of this cluster group revealed six sub-clusters that we identified by expression of *MMR1L4*, MHC II genes and *MMP9* as five sub-clusters comprising mainly monocytes and one comprising mainly heterophils. Chicken spleen macrophages have previously been divided into two groups by cell surface expression of MMR1L4 and MHC II, i.e., MMR1L4^high^MCHII^low^ and MMR1L4^low^MHCII^high^, respectively [32]. These populations were also assigned functional differences where MMR1L4^high^MCHII^low^ cells showed higher phagocytic capacity, higher migratory capacity, lower antigen presenting properties and lower expression of some pro-inflammatory cytokines compared to MMR1L4^low^MHCII^high^ cells. For circulating monocytes, we have also observed alterations in cell surface expression of MMR1L4 and MHC II upon bacterial infection of chickens with a rapid, transient shift towards MMR1L4^high^MCHII^low^ expressing monocytes early after infection [33, 34]. In the current data, we defined monocyte sub-clusters 0 and 1 as “MMR1L4 high” and sub-clusters 3–5 as “MMR1L4 low”. Conversely, sub-clusters 0 and 1 were generally “MHC II low” and sub-clusters 3–5 were generally “MHC II high” although the relative expression of MHC II varied more between the individual subclusters compared to the expression of *MMR1L4* that showed a more uniform level of expression within the two sub-cluster groups. Furthermore, differences in phenotype and functions between the monocyte sub-clusters could be inferred from expression of other immune genes. For example, for cells in “MMR1L4 high” sub-clusters high expression of *PPARG*, associated with M2-macrophages in mammals [35], and *S100A12* and *SPINK2* that have antimicrobial properties [36–38], were observed. For cells in “MMR1L4 low” sub-clusters several genes associated with cell activation and inflammatory responses were highly expressed e.g., *ALCAM* (CD166), *LOC39551* (CCL4), *SAA* and *TLR7*. Hence, the indicated functional differences between monocyte sub-clusters showed resemblance with the functional differences observed for chicken MMR1L4^high^MCHII^low^ and MMR1L4^low^MHCII^high^ spleen macrophages [32]. Moreover, our analysis indicated that chicken monocytes might have more functional subsets within the two general “MMR1L4 high” and “low” populations. This would be in analogy with mammalian systems where e.g., for humans three major and potentially several more minor different functional subsets of circulating monocytes have been identified [39].

For cells in sub-cluster 2 a high expression of *MMP9* and very low expression of *MMR1L4* and MHC II genes, were observed. It has previously been shown that *MMP9* is expressed in chicken heterophils but not in chicken monocytes [40]. Cells in subcluster 2 also showed a high expression of *BHLHE40* and *NR4A3* that in mammals have been associated with neutrophils [41, 42], i.e., the mammalian homologue to chicken heterophils. We therefore concluded that subcluster 2 likely contained mainly heterophils. In the primary analysis, the heterophils were embedded within monocyte cluster 6. This is likely due to their common origin as myeloid cells and many common functions, e.g., phagocytosis, resulting in similar general mRNA expression patterns that made the sub-cluster analysis necessary to distinguish between the two cell types. Interestingly, cells in the heterophil sub-cluster showed a striking “pro-inflammatory” mRNA-expression profile with high expression of e.g., *IL1B*, *IL18*, *CD83*, *TLR4*, and *TRAF3*. This could reflect a heterophil “high alert state” as sentinel cells with mRNA-expression of pre-formed cytokines in granules or secretory vessels for rapid release upon challenge, as suggested for mammalian neutrophils [43].

### T-cells

The identification of different T-cell populations in the current data set proved more challenging than that of B-cells and monocytes. Such problems have also been reported in other studies of single-cell RNA transcriptomics of T-cell subpopulations, for mouse and human T-cells [22], as well as for horse PBMC [15] and chicken spleen cells [18]. This can be due to limited resolution due to insufficient sequencing depth or to differences between mRNA expression levels and protein expression of markers by which T-cell populations are currently defined, e.g., CD4, CD8α and CD8β, or more probably a combination of both. We found that the expression of CD4, CD8α and CD8β never comprised all cells in a cluster. Similarly, very low expression of CD4 and CD8α was reported for chicken spleen cells [18]. Nonetheless, we were able to further identify the clusters comprising T-cells into: TCRγ/δ + T-cells, CD4 + cells, CD8αβ + cells and “cytolytic cells”.

Chicken TCRγ/δ+ T-cells have so far been divided into three main subpopulations based on their cell surface expression of CD8, i.e., TCRγ/δ+CD8-, TCR γ/δ+CD8αβ + and TCRγ/δ+ CD8αα+ [12]. The TCR γ/δ+CD8- subpopulation is usually dominant among PBMC, which was also the case for the cells used in the present study: approximately 94% TCR γ/δ+CD8-, 4% TCRγ/δ+ CD8αβ+ and 2% TCRγ/δ+ CD8α+ out of the whole TCRγ/δ+ population. By mRNA-expression we identified the majority of TCRγ/δ+ T-cells in the distinct group formed by clusters 1, 0, 23 and 25 that showed no expression of CD8. For TCRγ/δ+ T-cells in sub-cluster 2 of the cytolytic cells some CD8α expression was detected. The TCRγ/δ+ T-cells in the small cluster 29 showed no CD8 expression but because CD8 mRNA expression was generally low in the current dataset one may speculate that cluster 29 cells still comprised some TCRγ/δ+ CD8 + T-cells based on their close proximity to cluster 7 containing CD8αβ+ cells. Hence, it seems the group of clusters 1, 0, 23 and 25 comprised the TCRγ/δ+CD8- subpopulation, while cytolytic cell sub-cluster 2 and possibly cluster 29 comprised the TCRγ/δ+CD8 + subpopulations, which correlates well with the relative distribution of TCRγ/δ+ T-cell subpopulations detected by cell surface protein expression. Compared to the other TCRγ/δ+ T-cell clusters, cells in cluster 1 showed a high mRNA expression of *TPT1* that is considered important for TCR-mediated cell proliferation [44], and of ribosomal proteins and the cytokine *KK34*. We therefore concluded cells in cluster 1 comprised “activated” TCRγ/δ+ CD8- T-cells. Expression of the chicken TCRγ/δ+ T-cell specific cytokine *KK34* [45] was found in the group of clusters 1, 0, 23 and 25 but not in cluster 29 or cytolytic subcluster 2. Similarly, *KK34* expression was shown by single-cell RNA-seq for cells in the TCRγ/δ+ CD8- cluster, but not in the TCRγ/δ+ CD8 + cluster, of chicken spleen cells [18]. Cells in cluster 29 showed a distinct high expression of transcription factor *MYB* (c-Myb) that was not seen in any other T-cell cluster. In addition to its role in early TCRγ/δ+ T-cell development [46], *MYB* expression in mammalian peripheral T-cells has been found involved in e.g., proliferation [47], memory T-cell formation and T-cell exhaustion [48]. Cells in the group of clusters 1, 0, 23 and 25 shared a high expression of transcription factors *MAF*, *SOX13* and *GATA3* that was not evident in the other TCR γ/δ+ T-cell clusters. Of these, *MAF* (c-MAF) and *SOX13* have been identified as essential for the IL-17 producing TCRγ/δ+ T-cell subtype in mice [49, 50]. In mammals, GATA3 has several recognised functions in T-cells and T-cell development, e.g., as master regulator of Th2 differentiation and in regulation of group 2 innate lymphoid cell development and function [51]. Interestingly, a study of porcine TCRγ/δ+ T-cells showed that GATA3 protein expression correlated with the CD2- phenotype of TCRγ/δ+ T-cells, which was also characterised as CD8α^−/dim^CD27+perforin- while a subset of CD2 + TCRγ/δ+ T-cells were mainly GATA3- and T-bet + CD8α^high^CD27^−/dim^perforin+ [52]. In that study it was also suggested that these two TCRγ/δ+ T-cell subtypes represent different TCRγ/δ+ T-cell lineages and that CD2-GATA3 + CD8α^−/dim^CD27+perforin- TCRγ/δ+ T-cells may recognise antigen in a TCR-independent manner. Hence, the current results on mRNA expression profiles of chicken TCRγ/δ+ T-cells were likewise indicative of at least two distinctive subpopulations, i.e., the group of clusters 1, 0, 23 and 25 vs. cluster 29 and cytolytic subcluster 2, where the differences in transcription factor expression could indicate lineage differences. Moreover, cells in cluster 29 but not those in the other TCRγ/δ+ T-cell clusters, show expression of CD28 that is closely linked to TCR activation. This could thus indicate that the *GATA3* expressing cells in clusters 1, 0, 23 and 25 could be TCRγ/δ+ T-cells employing TCR-independent antigen recognition in analogy with what was suggested for porcine GATA3 + TCRγ/δ+ T-cells. Our observation on CD28 expression by TCRγ/δ+ T-cells was also supported by an early observation on cell surface protein expression, that some chicken TCRγ/δ+ CD8 + T-cells were CD28 positive while the remaining TCRγ/δ+ T-cells were CD28 negative [53].

We identified ten clusters comprising CD4 expressing cells that formed their own cluster group. Like the group of B-cell clusters, for most of these we were not able to distinguish between their mRNA-expression patterns to designate their identity or function beyond CD4 + T-cells. However, cells in cluster 14 showed a high mRNA expression of TCR subunits *CD3D* and *CD3E*, as well as of *TPT1* [44] and of ribosomal proteins. We therefore concluded cells in cluster 14 comprised “activated” CD4 + T-cells. Moreover, some cells in cluster 8 showed a high expression of *IL2RA* (CD25) and *CTLA4*, genes that in resting mammalian CD4 + T-cells are primarily expressed in Treg [54]. We therefore putatively identified cluster 8 as comprising Treg among other CD4 + T-cells. However, a hallmark of mammalian Treg is the expression of transcription factor Foxp3 that was recently also identified in the chicken [55]. The chicken Foxp3 gene was still missing from the reference annotation of the current chicken genome, and we therefore used additional methods to test for its expression but nevertheless could not detect this in the current dataset. This could be due to e.g., limited resolution, but consequently we currently lack definitive evidence for Treg in cluster 8. Interestingly, cells in cluster 11 showed a distinct high expression of *SMAD7* that in mice and humans have been associated with Th1 CD4+ T-cells involved in autoimmunity [56, 57].

We identified two clusters comprising CD8αβ+ T-cells, cluster 7 and cytolytic cell subcluster 1. Cells in cluster 7 did not show expression of genes associated with cytolytic activity, i.e., *GNLY*, *FASLG*, *GZMA* and *GZMM*, and we therefore concluded this cluster comprises resting and/or “non-effector/non-cytolytic” CD8αβ+ T-cells. In addition, some expression of *TARP* was also recorded for cells in cluster 7 and it is hence likely that this cluster contains a mixture of T-cells. The gene with highest expression in cluster 7 was *CD226* and this gene also showed a high expression in cytolytic cell subcluster 1. In humans and mice CD226 has a high cell surface expression on NK-cells and CTL [58] and our current results thus suggest that it might be a CTL marker in the chicken.

### Cytolytic cells

Cluster 4, positioned on its own near the other T-cell clusters, was found to contain a mixture of cell types and due to a high expression of genes associated with cytolytic activity, i.e., *GNLY*, *FASLG*, *GZMA* and *GZMM*, we identified it as comprising cytolytic cells. It appears that distinguishing different cell types with cytolytic functions can be challenging using single-cell RNA-seq as earlier described e.g., for horse PBMC [15]. By further analysis of cluster 4 we identified three subclusters putatively identified as mainly NK-cells in subcluster 0 (high expression of *CD247* (CD3ζ) [59]) and low expression of *CD3D*, *CD3E*, *TARP* and *TRBV6-5*), mainly CTL in subcluster 1 (high expression of *TRBV6-5* (TCRβ), *CD8A* and *CD8BP*) and mainly TCRγ/δ+ CD8+ T-cells in subcluster 2 (high expression of *TARP*). Interestingly, the relative expression of *GNLY*, *FASLG*, *GZMA* and *GZMM* showed different patterns for these cytolytic cell types, which could indicate cell type differences in preferential killing mechanisms. It should however be noted that to our knowledge, the hens used in the current study were not experiencing any infections and the cytotoxic cells should probably be regarded as “resting”. Hence, the expression of cytolytic activity genes might well be different when the cells are engaged in killing. For the TCRγ/δ+ CD8+ T-cells in subcluster 2 a high expression of *IL2RB* was noted, which corresponds well with the observation that spontaneous cytotoxicity of chicken spleen TCR γ/δ+CD8 + T-cells was enhanced by preincubation with interleukin-2 [60].

## Conclusions

Taken together, using single-cell RNA-seq we were able to identify most major leukocyte types with reasonable certainty and the results corresponded well with those obtained using conventional flow cytometric leukocyte phenotyping. Among B-cells we identified a subpopulation of putative terminally differentiated B-cells that are a novel observation among chicken leukocytes. For monocytes and TCRγ/δ T-cells we were able to identify and verify functional aspects of previously observed subpopulations as well as making novel observations. In depth identification of other T-cell subtypes proved more challenging although some new observations were made e.g., regarding cytotoxic cell types. This methodology definitively has benefits for cell identification and functional analysis in a species like the chicken where species specific reagents for classical immunological analyses are limited and it will further improve with a better annotation of the chicken genome. Data from the current study will probably provide more novel information when more knowledge on mRNA expression in chicken leukocytes is obtained, e.g., by transcriptomic analysis of purified cell populations and of cells during different immunological responses.

## Methods

### Chickens, sample collection and leukocyte preparation

Four clinically healthy 24-week-old Dekalb White Leghorn-type layer hens were sampled for this study. The hens were reared at a conventional pullet producer and moved to the high biosecurity animal facilities at the Swedish Veterinary Agency, Uppsala, Sweden, two weeks prior to sample collection. They belonged to a larger group of 15 hens and 3 cocks used for SPF-egg production and blood donation and were group housed in a pen with chopped straw on the floor, perches, laying nests, dust baths and water and feed *ad libitum* in a room with negative pressure ventilation. At the time of sampling some hens in the group had commenced laying but it was not known if the individuals sampled had laid eggs yet. Blood was collected from the jugular vein into sterile heparinised blood collection tubes (#368,494, BD Vacutainer ®, BD Life Sciences). The blood was diluted 1:1 in sterile phosphate buffered saline without Ca^2+^ and Mg^2+^ at pH 7 (PBS) and peripheral blood leukocytes were isolated by Ficoll-Paque PLUS (GE Healthcare Life Sciences) gradient centrifugation as previously described [61]. Cells were suspended in PBS supplemented with 2% foetal bovine serum (Gibco® #10,082,147, ThermoFisher Scientific) and 1 mM EDTA and the number of thrombocytes in the samples were reduced by immunomagnetic cell separation using the EasySep PE Positive Selection Kit II (#17,684, StemCell Technologies) and PE-conjugated mouse monoclonal antibody to chicken CD41/61 (clone 11C3, #AM05550PE-N, OriGene) according to the StemCell Technologies depletion protocol no. 28,898 as previously described for chicken leukocytes [61]. The depleted cell preparations were suspended in PBS with 0.04% bovine serum albumin (#A7030, Sigma-Aldrich), viability of cell preparations was estimated by trypan blue exclusion to 92–97%.

### Library preparation and sequencing

Cell samples were submitted within 3.5 h of blood sample collection to the Science for Life Laboratory SNP&SEQ Technology Platform, Uppsala, Sweden for library preparation and sequencing. Preparation of libraries from approximately nine thousand cells per bird was performed from fresh purified leukocytes with the Chromium NextGEM Single Cell 3′ v3.1 kit (10x Chromium), resulting in successful preparations from approximately five thousand cells per bird. Sequencing was performed using a NovaSeq SP flow cell (Illumina) to an average target depth of 35,000 reads/cell. Two lanes were used for each sample.

### Read counting

Read counting was performed using the 10x genomics software Cell Ranger [62] (Version: 7.0.0) standard workflow with the existing reference genome and genome annotation for the chicken (NCBI taxa: 9031. Assembly: RefSeq GCF_016699485.2; GenBank GCA_016699485.1). The used genome assembly was from 2021 and was assembled by the Vertebrate Genomes Project. The annotation (annotation release ID: 106) used was from 2021/2022.

Read counting was performed for each sample using Cell Ranger *count*, with the resulting matrix files used for downstream analysis. The computational pipeline includes read trimming, genome alignment using STAR (Spliced Transcripts Alignment to a Reference) [63], transcriptome alignment of reads based on transcriptome compatibility of mapped reads, Barcode correction, UMI counting and cell calling based on the EmptyDrops method [64, 65].

### Data preparation and integration

Putative doublets were removed from the matrices using the *DoubletDetection* [66] package in python. After doublet prediction the barcode list was edited to remove putative doublets.

Each matrix, barcode, and feature file set were loaded into R (Version: 4.2.2) as a *Seurat* [67] (Version: 4.3.0) object. Cells with a higher mitochondrial percentage than 20%, cells with a lower feature count than 300, and features that appear in less than three cells were filtered out from each object. RBCs were also filtered out based on HB-gene expression; cells with more than 5% HB genes were filtered out. After filtering, the data were normalised in *Seurat* using *SCTransform()* independently on each of the four objects with method “*glmGamPoi*”.

The data was integrated on 3000 variable features that were prepared using *PrepSCTIntegration()* and calculated using *SelectIntegrationFeatures()* on the four Seurat objects. Anchor cells were defined using *FindIntegrationAnchors()* with normalization.method set to *“SCT”*. The anchors were then used for integration using *IntegrateData()* with normalization.method set to *“SCT”.*

### Cluster determination

Dimensionality reduction was performed using PCA on the variable features calculated for the data integration. The cumulative proportion of variance was calculated and used to determine how many principal components (PCs) to keep. The cut-off was set so that 90% of variance would be conserved; this resulted in 27 PCs being used for downstream analyses for the complete dataset.

After PCA, the nearest neighbours for each cell were computed using *FindNeighbors()* on the first 27 dimensions. *FindClusters()* was called to establish cluster boundaries using the standard Louvain algorithm. The resolution was evaluated using *clustree* [68]. The default resolution of 0.8 was used when analysing the entire dataset and different resolutions were evaluated and used when re-analysing subsets of data.

For cluster visualisation UMAP was run using *RunUMAP()* with reduction set to *“pca”* on the first 27 dimensions.

### Cluster annotation

Annotations of the clusters were performed using a combination of manual annotation by inspection of differentially expressed genes and GO term enrichment.

The marker gene-based annotation was performed by sub-setting the cells by cluster identity and running the *FindMarkers()* function in on these cells with assay set to *“SCT”* and only.pos set to *TRUE*.

GO- and KEGG-term enrichment analysis was performed using the R-library gprofiler2 [69]. The GO terms were created per cluster using *gost()* with the top 100 differentially expressed genes as the query. The GO search was performed against the reference database for *Gallus gallus*.

Potential expression of recently discovered chicken *FOXP3* [55] that was still missing from the reference annotation of the chicken genome was tested both by manually adding the putative *FOXP3* gene to the annotation file before read counting and looking for expression (in one sample) and by using short nt blast on a blast database constructed of all reads per sample and querying the mRNA sequence against this database. Neither approach showed any indication of expression of *FOXP3* in the current samples.

### Re-analysis

The major clusters were all re-analysed using the above steps on the corresponding cell subsets. This was done to investigate if any biologically significant subgroups could be found within the original clusters.

### Immunofluorescence labelling of purified leukocytes

Immunofluorescence labelling and flowcytometric analysis was used to identify leukocytes according to cell surface expression of lineage markers as previously described [70]. In brief, samples of purified leukocytes were labelled with either of the three antibody panels listed in Additional file 5 for 20 min at room temperature in the dark, panel 1 was used on samples both prior and after immunomagnetic separation while panels 2 and 3 were only used with depleted cell samples. LIVE/DEAD® fixable Aqua dead stain (#L34957, ThermoFisher Scientific) was used for dead cell exclusion in all panels. Cells were subsequently washed and fixed with 1.25% paraformaldehyde and stored at 4 °C until analysis.

Flow cytometry was performed using a BD FACSVerse (BD Biosciences), equipped with 488 nm blue, 633 nm red and 405 nm violet lasers and results were analysed using the FACSDiva (BD Biosciences) software. Single-stained compensation controls and fluorescence minus one (FMO) negative controls were included in the assays, the gating strategies are shown in Additional file 6 and samples were recorded until 30,000 events in the CD45 gate were acquired. Titrations of all antibodies were performed to determine optimal labelling conditions prior to the experiment.

### Electronic supplementary material

Below is the link to the electronic supplementary material.

Supplementary Material 1

Supplementary Material 2

Supplementary Material 3

Supplementary Material 4

Supplementary Material 5

Supplementary Material 6

## Data Availability

RNA sequencing datasets generated in the current study are available in the Gene Expression Omnibus (ncbi.nlm.nih.gov/geo) under accession number GSE224329 and the Sequence Read Archive (ncbi.nlm.nih.gov/sra) under accession numbers SRX19259178, SRX19259179, SRX19259180, and SRX19259181. All R scripts are available at https://github.com/maxwelma/exjobb. All results on leukocyte phenotyping and thrombocyte depletion are included in this published article and raw data are available from the corresponding author on reasonable request.
