# Supplementary material for: Single-cell RNA-seq mapping of chicken peripheral blood leukocytes
Source: BMC Genomics. 2024 Jan 29;25:124. doi: 10.1186/s12864-024-10044-4 (PMC10826067; doi:10.1186/s12864-024-10044-4)
Supplement: Supplementary file 1 — Supplementary Material 1 [file 12864_2024_10044_MOESM1_ESM.pdf]

Additional file 1. Outcome of immunomagnetic depletion of CD41/61+ cells and identification of chicken leukocyte populations by immunofluorescence labelling and flow cytometric analysis of cell preparations used for single cell RNA-seq

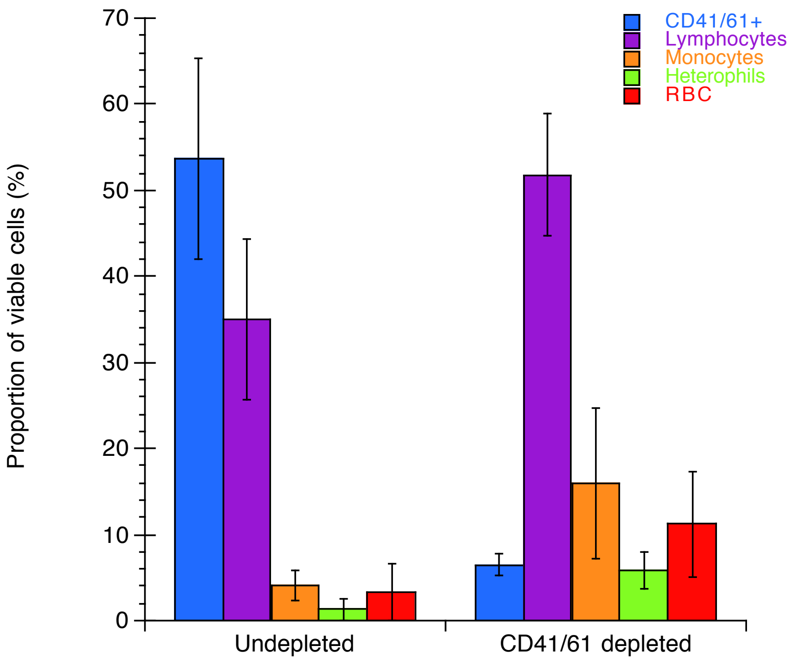

Additional figure 1. Proportions of CD41/61+ cells (thrombocytes; blue), lymphocytes (purple), monocytes (orange), heterophils (green) and red blood cells (RBC; red) as defined by immunofluorescence labelling and flow cytometric analysis of chicken PBMC before and after immunomagnetic depletion of CD41/61+ cells (means±95% confidence intervals, n=4).

Additional table 1. Proportions of lymphocyte subpopulations identified by immunofluorescence labelling and flow cytometry in chicken PBMC before and after immunomagnetic depletion of CD41/61+ cells (means±95% confidence intervals, n=4).

Subpopulation	Undepleted PBMC (%)	CD41/61 depleted PBMC (%)
Bu-1+ (B-cells)	6.4±2.9	12.2±5.4
TCRα/Vβ1+	15.1±2.4	15.8±4.6
TCRα/Vβ2+	n.t.	5.4±1.1
<u>CD4+ (all)</u>	n.t.	12.1±1.7
CD4+CD8αα+	n.t.	1.5±1.1
CD4+CD25+	n.t.	0.77±0.24
CD8α+ (all)	n.t.	9.8±6.8
CD8β+ (all)	n.t.	7.4±4.2
<u>TCRγ/δ+ (all)</u>	n.t.	18.4±8.5
TCRγ/δ+CD8-	n.t.	17.2±7.7
TCRγ/δ+CD8αα+	n.t.	0.38±0.35
TCRγ/δ+CD8αβ+	n.t.	0.78±0.59
TCRγ/δ-CD8αβ+	n.t.	6.1±3.4

n.t. – not tested

Proportions are out of viable cells identified in the gating strategies shown in Additional file 6, the antibody panels used are described in Additional file 5 and the labelling protocol and flow cytometric analysis are described in Materials and methods.
