# Supplementary material for: Single-cell RNA-seq mapping of chicken peripheral blood leukocytes
Source: BMC Genomics. 2024 Jan 29;25:124. doi: 10.1186/s12864-024-10044-4 (PMC10826067; doi:10.1186/s12864-024-10044-4)
Supplement: Supplementary file 2 — Supplementary Material 2 [file 12864_2024_10044_MOESM2_ESM.pdf]

Additional file 2. Monocyte clusters 5 and 6

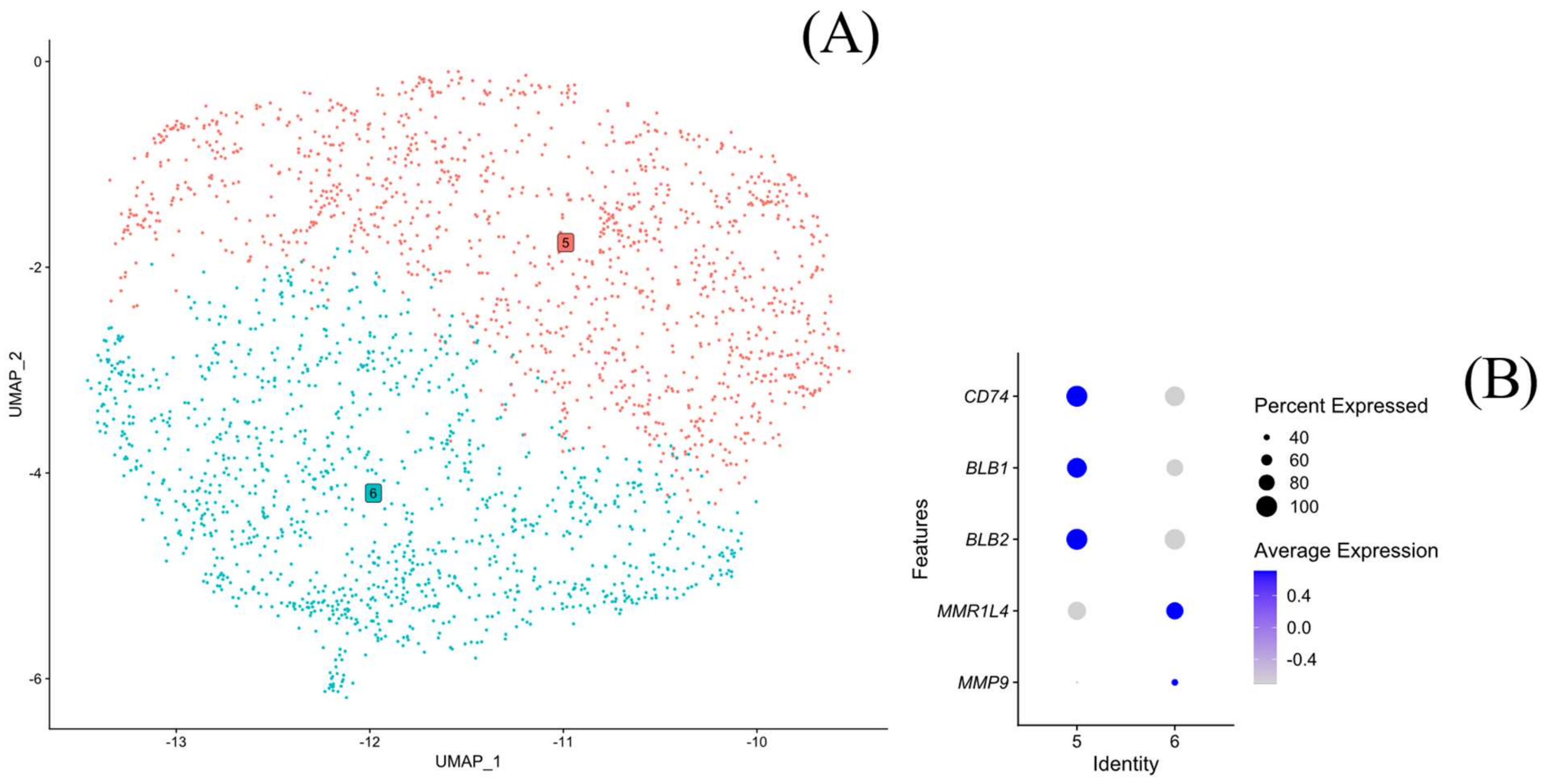

Additional file 2. (A) UMAP of monocyte clusters 5 and 6 (MMR1L4+ cells). B) Dot plot of expression of a selection of MHCII genes (CD74, BLB1 and BLB2), mannose receptor MMR1L4 and MMP9 in the indicated clusters. The radius of the dot corresponds to percentage of cells in each cluster expressing the gene, and colour intensity corresponds to scaled expression values. Expression values are scaled within the plot.
