# Supplementary material for: Single-cell RNA-seq mapping of chicken peripheral blood leukocytes
Source: BMC Genomics. 2024 Jan 29;25:124. doi: 10.1186/s12864-024-10044-4 (PMC10826067; doi:10.1186/s12864-024-10044-4)
Supplement: Supplementary file 3 — Supplementary Material 3 [file 12864_2024_10044_MOESM3_ESM.pdf]

Additional file 3. Cluster 22 proliferating cells

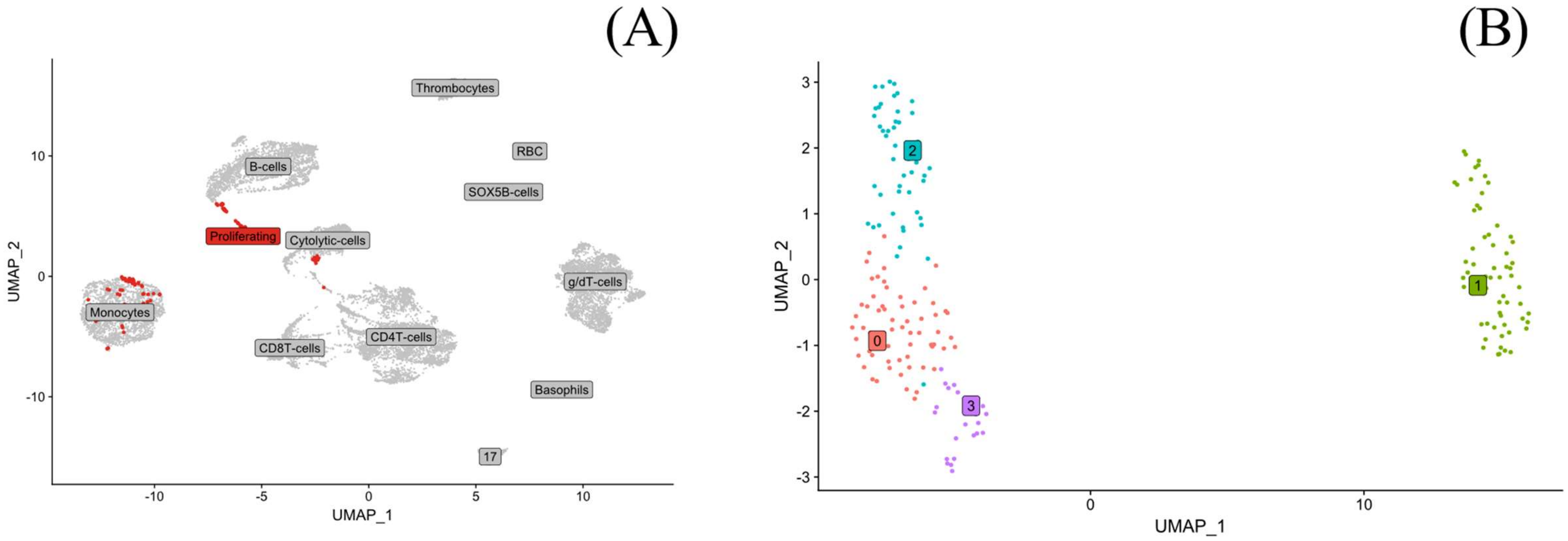

Additional file 3. (A) UMAP of the 16 936 studied cells with cells proliferating cells in cluster 22 indicated in red. (B) UMAP after re-clustering cells of proliferating cells in initial cluster 22 with putative cell types annotated based on the differential expression within the subcluster. The analysis identified four subclusters 0-3 identified by different colour cells
