# Supplementary material for: Single-cell RNA-seq mapping of chicken peripheral blood leukocytes
Source: BMC Genomics. 2024 Jan 29;25:124. doi: 10.1186/s12864-024-10044-4 (PMC10826067; doi:10.1186/s12864-024-10044-4)
Supplement: Supplementary file 4 — Supplementary Material 4 [file 12864_2024_10044_MOESM4_ESM.pdf]

Additional file 4. Identification of chicken leukocytes immunofluorescence labelling and by single-cell mRNA expression

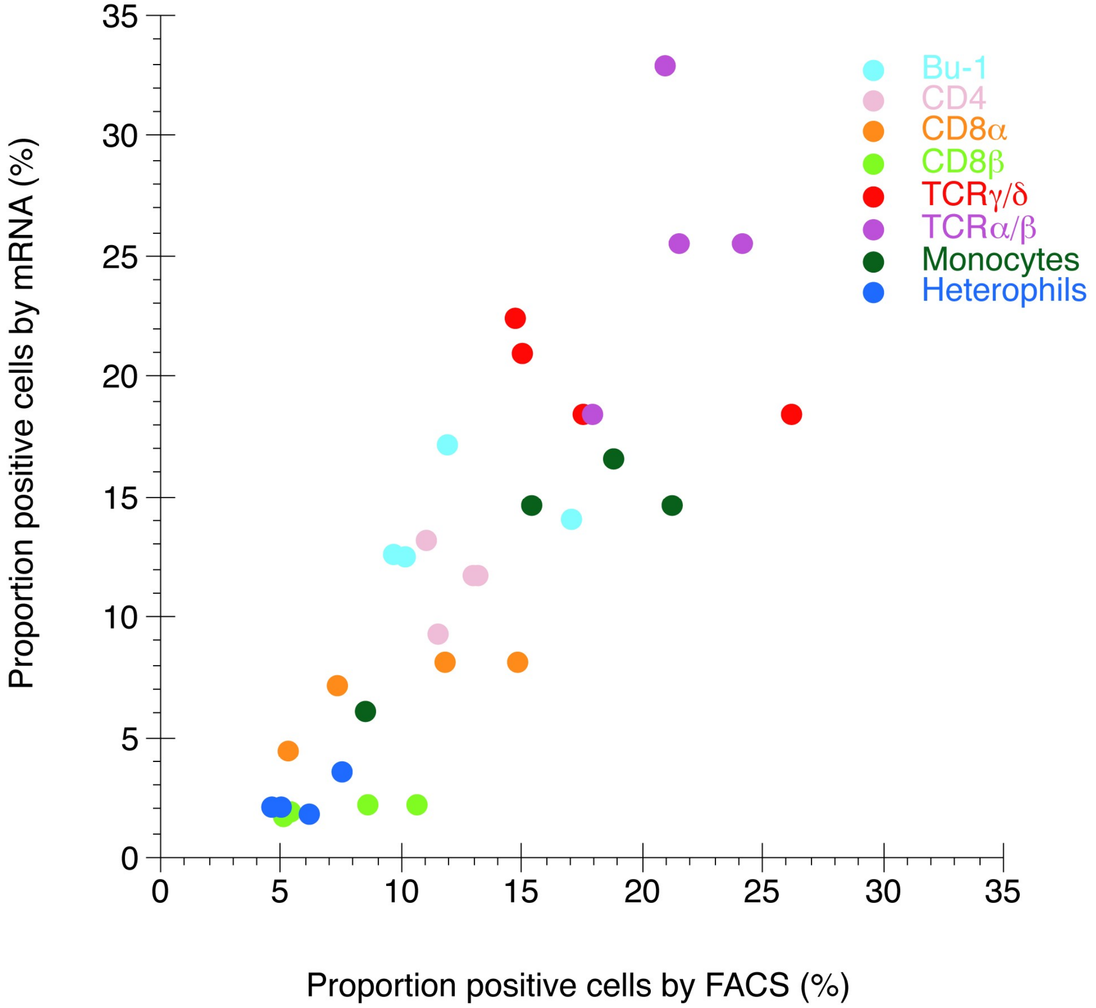

Additional file 4. Identification of chicken leukocytes in the indicated populations by phenotypical identification by immunofluorescence labelling and flow cytometric analysis (FACS) and by single-cell mRNA expression (mRNA) in leukocyte preparations from the four individual hens. For phenotypical identification, cells were identified as cells in the "lymphocyte gate" with cell surface expression of Bu-1, CD4, CD8α, CD8β, TCRγ/δ and TCRα/β (combination of TCRα/$V\beta_1$ and TCRα/$V\beta_2$), respectively. Monocytes were identified by FSC/SSC characteristics and cell surface expression of MMR1L4 (MRC1L-B) and heterophils were identified by FSC/SSC characteristics and cell surface expression of CD45. Data was expressed as proportions of populations out of live events for each individual hen. For gating strategies see Additional file 6. For mRNA identification, cells with log^2 fold change ≥0.25 expression of *LOC396098* (Bu-1), *CD4*, *CD8A* (CD8α), *CD8BP* (CD8β) and *MMR1L4* were considered positive. For TCRγ/δ, cells in clusters 1, 0, 23, 25 and 29 were considered positive, for TCRα/β cells in clusters 3, 8, 11-16, 24, 30 and 7 were considered positive and for heterophils cells in monocyte subcluster 2 were considered positive. Data was expressed as proportions of positive cells out of the total number of cells after filtering for each individual hen.
