# Supplementary material for: Single-cell RNA-seq mapping of chicken peripheral blood leukocytes
Source: BMC Genomics. 2024 Jan 29;25:124. doi: 10.1186/s12864-024-10044-4 (PMC10826067; doi:10.1186/s12864-024-10044-4)
Supplement: Supplementary file 5 — Supplementary Material 5 [file 12864_2024_10044_MOESM5_ESM.pdf]

Additional file 5. Monoclonal antibodies used for immunolabelling and combinations (panels) used to phenotype chicken leukocytes.

Abbreviation	Clone	Specificity	Fluorochrome	Panel		
				1	2	3
CD41/61-RPE	11C3	Chicken CD41/61 intergrin (GPIIb-IIIa)	R-phycoerythrin[d]	X	X	X
CD45-PerCp/Cy5.5	UM16-6[b]	Chicken CD45, all isoforms [1]	Peridinin chlorophyll-cyanine 5.5[e]	X	X	X
MRC1L-B	Kul-01[c]	Chicken mannose receptor MMR1L4 (MRC1L-B) [2]	Alexa Fluor 647[d]	X	-	-
TCRα/Vβ_1-Fitc	TCR-2[c]	Chicken α/Vβ_1 T-cell receptor	Fluorescein[d]	X	-	-
Bu-1-PACBLU	AV20	Chicken Bu-1 (ChB6) alloantigen [3]	Pacific Blue™[d]	X	-	-
TCRα/Vβ_2-RPE	TCR-3[c]	Chicken α/Vβ_2 T-cell receptor	Fluorescein[d]	-	X	-
CD4-PACBLU	CT-4[c]	Chicken CD4	Pacific Blue™[d]	-	X	-
CD25-PE/Cy7	AV142[b]	Chicken CD25, interleukin-2 receptor α-chain	R-phycoerythrin-cyanine 7[e]	-	X	-
CD8α-Cy5	3-298[c]	α-chain of chicken CD8	Cyanine 5[d]	-	X	X
CD8β-APC/Cy7	EP42[c]	β-chain of chicken CD8	Allophycocyanin-cyanine 7[e]	-	X	X
TCRγ/δ-Fitc	TCR-1[c]	Chicken γ/δ T-cell receptor	Fluorescein[d]	-	-	X

X: used in panel, -:not used in panel
a Purchased from OriGene.
b Purchased from Bio-Rad Antibodies.
c Purchased from SouthernBiotech.
d Fluorochrome conjugated by manufacturer.
e Fluorochrome conjugated using Lightning-Link® conjugation kits (abcam) according to the manufacturer's protocol.

References

1. Huhle D, Hirmer S, Göbel TW (2017) Splenic γδ T cell subsets can be separated by a novel mab specific for two CD45 isoforms. Dev Comp Immunol 77:229-240 doi:10.1016/j.dci.2017.08.013
2. Staines K, Hunt LG, Young JR, Butter C (2014) Evolution of an expanded mannose receptor gene family. PLoS One 9 (11):e110330 doi:10.1371/journal.pone.0110330
3. Rothwell CJ, Vervelde L, Davison TF (1996) Identification of chicken Bu-1 alloantigens using the monoclonal antibody AV20. Vet Immunol Immunopathol 55 (1-3):225-234. doi:10.1016/s0165-2427(96)05635-8
