# Supplementary material for: Single-cell RNA-seq mapping of chicken peripheral blood leukocytes
Source: BMC Genomics. 2024 Jan 29;25:124. doi: 10.1186/s12864-024-10044-4 (PMC10826067; doi:10.1186/s12864-024-10044-4)
Supplement: Supplementary file 6 — Supplementary Material 6 [file 12864_2024_10044_MOESM6_ESM.pdf]

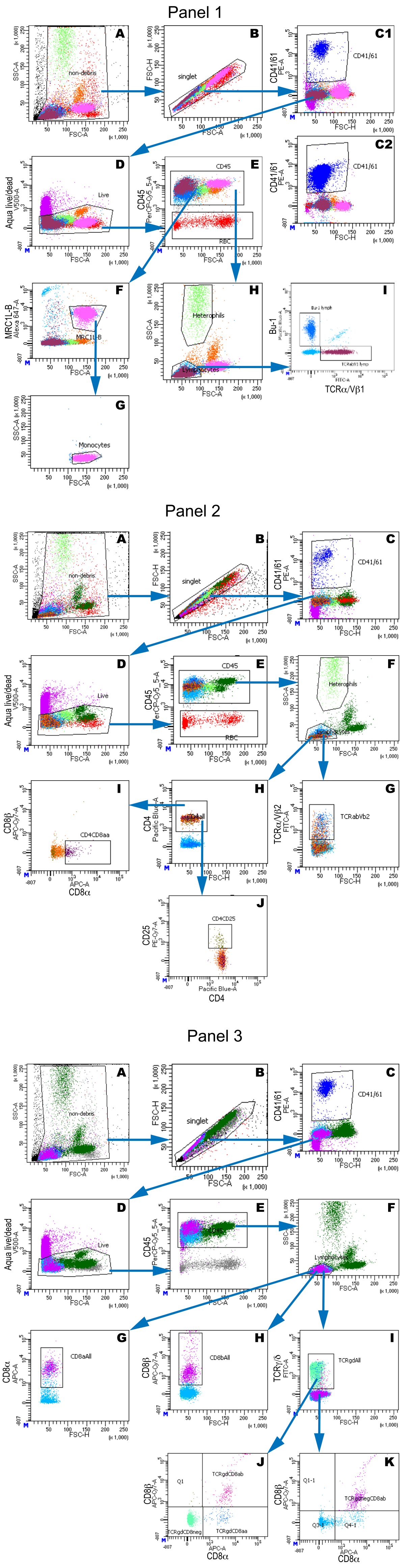

Additional file 6. Gating strategy for phenotypic identification of leukocytes with immunofluorescence labelling and flow cytometric analysis.

The antibody panels are listed in Additional file 5.

Panel 1.

Identification of CD41/61+ cells (thrombocytes), red blood cells, monocytes, heterophils, lymphocytes, TCRα/Vβ_1+ lymphocytes and Bu-1+ lymphocytes (B-cells), respectively, through singlet gating, FSC/SSC characteristics and using CD41/61-PE, CD45-PerCp/Cy5.5, MRC1L-B-AF647, TCRα/Vβ_1-Fitc and Bu-1-PACBLU. From the gate excluding debris in initial dot-plot in A gating through FSC-H vs FSC-A was performed in B to exclude large aggregates. CD41/61+ cells were identified in C. Due to high autofluorescence of paraformaldehyde fixed thrombocytes in the V500-channel these had to be excluded before identification of live cells. C1 shows a depleted cell preparation C2 shows the undepleted cell preparation from the same hen. From non-CD41/61+ cells in C live cells were gated through exclusion of events stained with Aqua dead stain in D. From the live gate, CD45 high events and CD45- events (red blood cells), respectively were gated in E. From the CD45 high events in E MRC1L-B+ events were gated in F and these were defined as monocytes according to FSC/SSC characteristics in G. CD45 high events in E were identified as heterophils and lymphocytes, respectively according to FSC/SSC characteristics in H. Lymphocytes gated in H were defined according to Bu-1 expression (B-cells) and TCRα/Vβ_1 expression respectively in I. A representative CD41/61 depleted leukocyte sample is shown.

Panel 2.

Identification of TCRα/Vβ_2+ lymphocytes, CD4+ lymphocytes, CD4+CD8αα+ lymphocytes and CD4+CD25+ lymphocytes, respectively, through singlet gating, FSC/SSC characteristics and using CD41/61-PE, CD45-PerCp/Cy5.5, TCRα/Vβ_2-Fitc CD8α-Cy5, CD8β-APCCy7 and CD4-PACBLU. A to E was analysed as described for panel 1 above. CD45 high events in E were identified as lymphocytes according to FSC/SSC characteristics in F. Lymphocytes gated in F were defined according to TCRα/Vβ_2 expression in G and according to CD4 expression in H (all CD4+ lymphocytes). CD4+ lymphocytes were defined as CD4+CD8αα+ in I and CD4+CD25+ in J, respectively. A representative CD41/61 depleted leukocyte sample is shown.

Panel 3.

Identification of TCRγ/δ+, CD8α+, CD8β+, TCRγ/δ+CD8-, TCRγ/δ+CD8αβ+, TCRγ/δ+CD8αα+ and TCRγ/δ-CD8αβ+ (CTL) lymphocytes, respectively, through singlet gating, FSC/SSC characteristics and using CD41/61-PE, CD45-PerCp/Cy5.5, TCRγ/δ-Fitc CD8α-Cy5 and CD8β-APCCy7. A to F was analysed as described for panel 2 above. Lymphocytes gated in F were defined according to CD8α expression in G (all CD8α+ lymphocytes), CD8β+ expression in H (all CD8β+ lymphocytes) and TCRγ/δ expression in I (all TCRγ/δ+ lymphocytes). TCRγ/δ+ lymphocytes gated in I were defined according to CD8α and CD8β expression, respectively, in J. TCRγ/δ- lymphocytes gated in I were defined according to CD8α and CD8β expression, respectively, in K. A representative CD41/61 depleted leukocyte sample is shown.
